# Design, development, and pilot study of a scale for educational professionals to assess theory of mind-related skills regarding children with ASD

**DOI:** 10.1371/journal.pone.0355044

**Published:** 2026-08-28

**Authors:** Carolina Messer-Soubelet, Bernardo Riffo, Omar Barriga

**Affiliations:** 1 Programa de Doctorado en Salud Mental, Facultad de Medicina, Universidad de Concepción, Concepción, Chile; 2 Departamento de Español, Facultad de Humanidades y Arte, Universidad de Concepción, Concepción, Chile Centro de Investigación Avanzada en Educación, CIAE, Concepción, Chile; 3 Departamento de Sociología, Facultad de Ciencias Sociales, Universidad de Concepción, Concepción Chile; University of the Basque Country UPV / EHU, SPAIN

## Abstract

**Background:**

Theory of mind (ToM) is a socio-cognitive capacity that enables individuals to attribute mental states to themselves and others and is increasingly recognized as relevant in educational settings, particularly when working with students diagnosed with autism spectrum disorder (ASD). However, most empirical work on ToM has focused on clinical populations, and little is known about how ToM-related skills are structured among education professionals. This article addresses this gap by developing and conducting a preliminary psychometric evaluation of a self-report instrument designed to assess ToM-related skills in professionals working with children diagnosed with ASD in school settings implementing inclusion-related policies.

**Methods:**

A cross-sectional study was conducted with 291 professionals from Chilean schools implementing inclusion-related policies. Participants completed a newly developed self-report questionnaire. Due to institutional participation rates, the final sample was classified as a convenience sample. An exploratory factor analysis (EFA) using maximum likelihood extraction and varimax rotation was conducted to examine the latent structure of the instrument. Sampling adequacy was supported by a Kaiser-Meyer-Olkin (KMO) coefficient of 0.91. Internal consistency was assessed using Cronbach’s alpha coefficients.

**Results:**

The EFA yielded a 35-item solution grouped into five factors, organized into two broad domains: “Identification of others’ mental states” (interpersonal dimension) and “Identification of one’s own mental states” (intrapersonal dimension). The interpersonal dimension emerged as a single factor encompassing emotion recognition, making inferences, and empathic response. The intrapersonal dimension was represented by four factors: awareness of one’s own thoughts and emotions, reflective ability, planning ability, and emotion regulation ability. All factors showed high internal consistency (Cronbach’s alpha > 0.8).

**Conclusions:**

This study provides preliminary evidence of the internal structure and reliability of a new self-report instrument assessing ToM-related skills in education professionals working with students diagnosed with ASD. Further validation in larger and more diverse samples is required before the instrument can be used to examine associations with classroom processes or other outcomes.

## Introduction

Theory of mind (ToM) is a fundamental concept in the field of social cognition, referring to the ability to attribute mental states to oneself and others, such as intentions, beliefs, and desires, thereby enabling the prediction of others’ behavior [1–4]. This cognitive capacity is crucial for understanding one’s own behavior and that of others and constitutes a cornerstone of social interaction and effective communication [5–8].

ToM is recognized as a complex and multidimensional construct that encompasses both cognitive and affective components. Cognitive ToM involves understanding that others may hold beliefs that differ from one’s own, whereas affective ToM refers to understanding others’ emotions and their interpersonal significance [9–11]. ToM can also be described in terms of interpersonal and intrapersonal dimensions. The interpersonal dimension involves recognizing and inferring the thoughts and emotions of others, whereas the intrapersonal dimension involves self-reflection on one’s own mental states and the regulation of emotional responses [10,11].

The relevance of ToM extends to educational contexts, where it has been linked to the development of meaningful social relationships and academic performance. Research indicates that the ability to understand false beliefs influences friendship development, especially among boys, whereas understanding emotions is associated with prosocial behaviors in girls [12,13]. Furthermore, ToM has been associated with improved reading comprehension, highlighting its role in cognitive and academic development [14–18].

In teachers, research on ToM has reported positive associations between mentalization and reductions in students’ aggressive behaviors, as well as improvements in academic achievement [19–21]. Mentalization has also been positively associated with well-being, lower levels of stress [22,23], the development of meaningful teacher-student relationships [24], and lower levels of burnout [25].

Despite its fundamental role, the application of ToM-related skills in real-life situations, particularly among education professionals working with children diagnosed with autism spectrum disorder (ASD), remains underexplored. Addressing this gap may help inform professional training and support the development of educational environments that facilitate the social-emotional development of students with ASD.

Existing instruments for assessing ToM focus primarily on clinical research settings and in many cases on populations with psychiatric disorders. Among the instruments designed to assess ToM in adolescents and adults are the “Mentalizing Stories for Adolescents” (MSA) [26], the “Reflective Functioning Scale” (RFS) [27], its later version, the “Reflective Functioning Questionnaire” (RFQ) [28], the “Metacognition Assessment Scale” (MAS) [27], the “Mentalization Questionnaire” (MZQ) [29], the “Mentalization Imbalances Scale” (MIS) [30], “The Mentalization Scale” (MentS) [31], and the “Multidimensional Mentalizing Questionnaire” (MMQ) [32].

These instruments are valuable for identifying deficits in individuals with neuropsychiatric disorders [33]. For example, the MSA has been used to examine differences between receptive language and mentalization [34]; the RFS has been used to assess parents’ ability to understand mental states [35]; the RFQ has been used to study the relationship between birth trauma and depression [36], as well as parental stress [37]; the MAS has been used to assess metacognitive profiles in patients undergoing psychotherapy [27], and in patients with schizophrenia [38]; the MZQ has been used in studies of patients with depression [39,40]; the MIS has been used in patients with personality disorders [41,42]; the MentS has been used in patients with psychosis [43] and anorexia [44]; and the MMQ has been used to evaluate memory in patients with Parkinson’s disease [45], and in studies of caregivers of patients with neurological disorders [46].

Other assessments used in adults include adaptations of the “Reading the Mind in the Eyes Test” (RMET) [47], the “Faux Pas Test” [48] and the “Empathy Quotient” (EQ) [49], which have been used to establish differences in mentalizing ability between neurotypical adults and adults diagnosed with ASD. In the Chilean context, the “Multidimensional Mentalizing Questionnaire” (MMQ) has been validated and operationalizes mentalization as a general self-reported disposition [50].

Despite the wide availability of instruments for assessing ToM in adults, recent systematic reviews have identified substantial heterogeneity in their psychometric quality, variability in the evidence supporting their validity, and important differences across assessment methods. This limits comparability and requires careful justification when ToM is examined as an applied construct [51–53]. Moreover, most available measures were developed for clinical purposes or general research and were not designed to capture the situated demands of educational practices, where professionals must identify socio-emotional cues in the classroom, anticipate students’ socio-emotional reactions, and make rapid, context-dependent adjustments. As a result, applying these instruments in school settings is likely to yield decontextualized measurements with limited sensitivity to situation-specific demands. This limitation is particularly evident in the instrument validated in Chile, which operationalizes mentalization as a general self-reported disposition and does not refer to its use in educational context [50]. Consequently, despite the extensive literature on ToM assessment, existing tools remain insufficient for evaluating how ToM is enacted in specific social contexts, particularly in school settings implementing inclusion-related policies. This highlights the absence of specialized measures for assessing ToM-related skills among teachers and education professionals working with students diagnosed with ASD. This gap is particularly relevant given the complexities of ASD, a neurodevelopmental condition characterized by difficulties in communication and social interaction [54,55], and the need for professionals to deploy contextually responsive socio-cognitive skills that traditional instructional approaches may not adequately support [3,56,57].

The Chilean education system, guided by the principles of inclusion and equal opportunity [58,59], offers a relevant context for examining ToM-related skills in educational practices. The School Integration Program (PIE, by its initials in Spanish) exemplifies this policy framework by promoting the principles of “Presence, Recognition, and Relevance” for students with special educational needs. PIE facilitates multidisciplinary collaboration among professionals from various fields, including special educators, psychologists, and speech therapists, among others, who together form the “integration team”. It also promotes curricular flexibility and provides additional financial support. In this way, PIE seeks to foster inclusive environments that support the diverse needs of all students [59–64].

Given that teachers and integration team professionals are assumed to have fully developed ToM-related skills, it is essential to assess how these skills are applied during interactions with children diagnosed with ASD. Such an assessment may help identify context-specific challenges in professional-student interactions and inform strategies to support educational practices in schools implementing inclusion-related policies.

As the current literature provides limited evidence on the application of ToM-related skills among neurotypical adults in professional educational roles, the use of these skills by teachers and integration team professionals in school settings implementing inclusion-related policies remains a critical area for research.

In view of this literature void, an instrument was developed as part of a broader project examining educational processes in schools implementing inclusion-related policies. The present article, however, focuses exclusively on the design and preliminary psychometric evaluation of this self-report instrument. Specifically, this work addresses the lack of measures tailored to educational practice by developing a self-report questionnaire to assess how teachers and integration team professionals apply ToM-related skills when working with students diagnosed with ASD. Such an instrument may help identify areas in which ToM-related abilities could be strengthened and, in the longer term, inform efforts to support educational environments that foster the social–emotional development of students with ASD. However, the instrument was not designed to measure ToM as a latent cognitive ability or performance-based construct. Rather, it assesses professionals’ self-reported ToM-related skills as applied to everyday interactions with students diagnosed with ASD in school settings.

Accordingly, this article presents the design, construction, and first pilot study of a self-report instrument aimed at assessing how education professionals, including teachers and members of integration teams, perceive and report their use of ToM-related skills in their day-to-day work with school-aged students diagnosed with ASD.

## Methods

**Fig 1:**
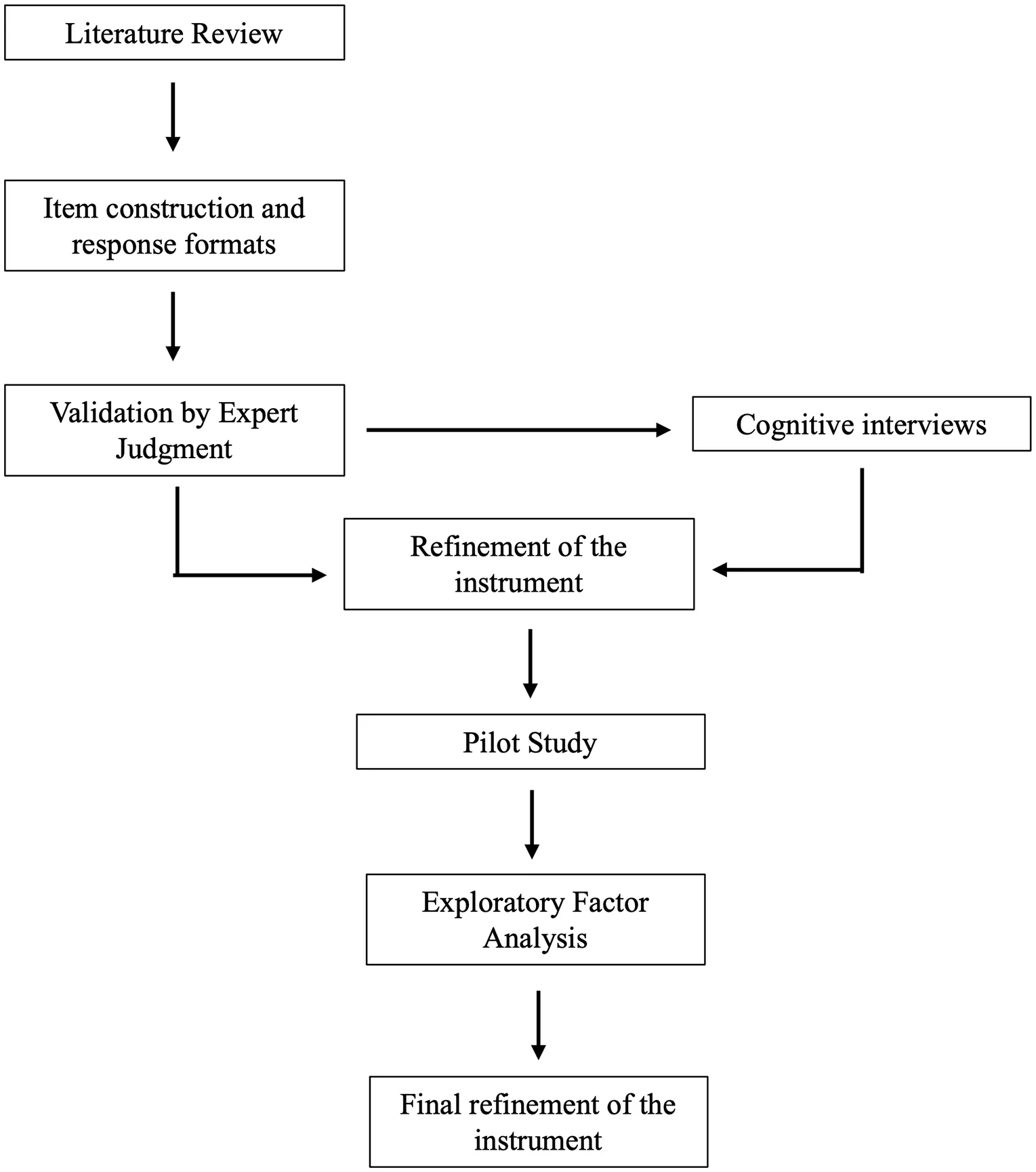
Stages of instrument design, development, and refinement. The process began with a literature review that informed the construction of the items and sections of the questionnaire. The instrument then underwent expert validation, followed by cognitive interviews with key informants. Feedback from these steps was used to refine the instrument in terms of the number of questions and the response format. A pilot study was subsequently conducted, and the results were used to perform an exploratory factor analysis that informed the final version of the instrument.

The construction, design, development, and refinement of the instrument were carried out in eight stages: (1) literature review, (2) construction of items and response formats, (3) validation by expert judgment, (4) cognitive interviews, (5) refinement of the instrument, (6) pilot study, (7) exploratory factor analysis, and (8) final refinement of the instrument (see Fig 1).

### Literature Review/ Item construction and response format

First, a literature review on ToM and its dimensions was conducted. Using Westby’s model [11] as a reference framework, together with contributions from the literature on affective and cognitive ToM, the skills to be assessed were identified. Subsequently, the emotions to be included in the questionnaire were selected using the Test of Emotion Comprehension (TEC) by Pons, Harris, and Rosnay [65] as a reference.

**Fig 2:**
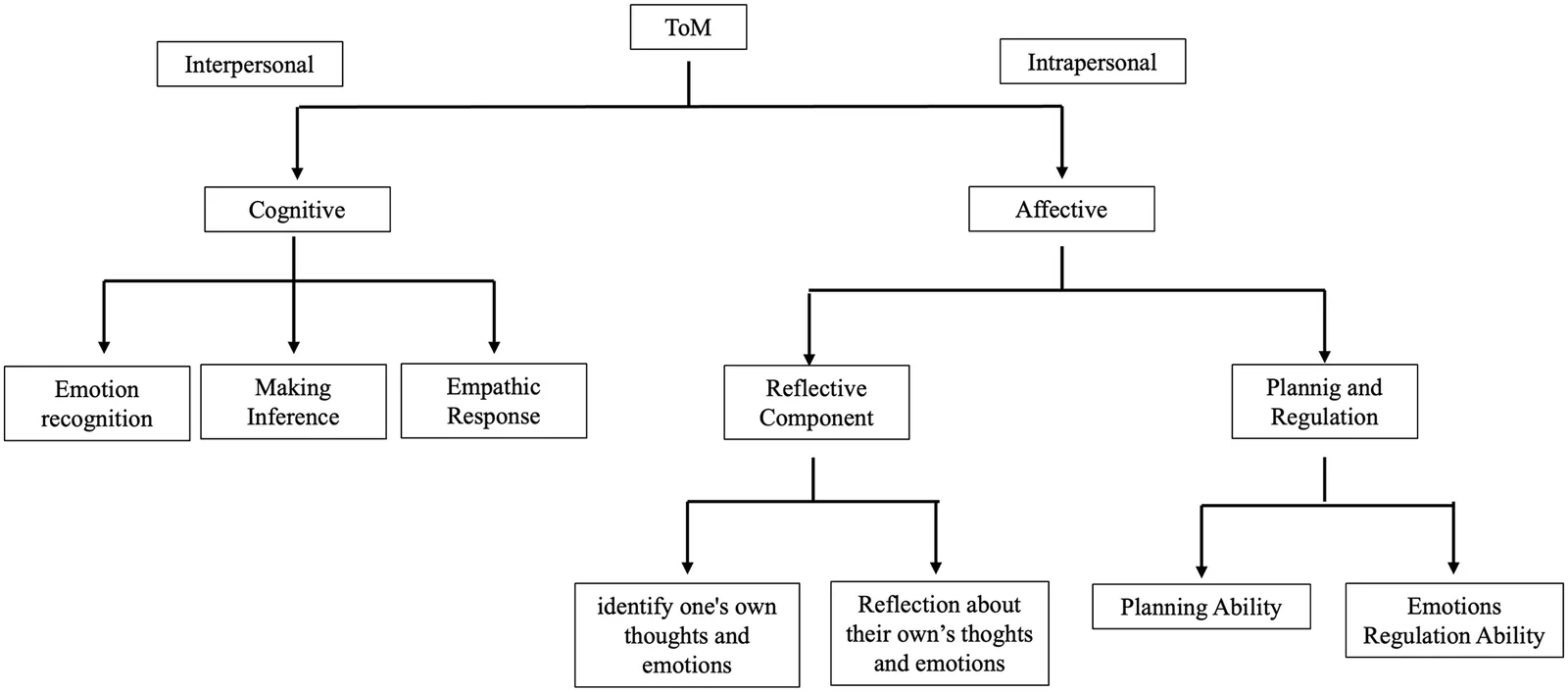
Westby’s ToM model adapted for the developed instrument. In the adapted ToM model, five sections of the instrument are identified. As part of the interpersonal dimension of ToM, the following are included: emotion recognition, making inferences and empathic response. For the intrapersonal dimension, a section called “reflective component” is included, which consists of the ability to identify one’s own emotions and thoughts and to reflect on them. In addition, planning and regulation are divided into two subsections: planning ability and emotion regulation ability.

Following these guidelines, five basic sections were defined: (1) emotion recognition, (2) making inferences, (3) empathic response, (4) reflective component, and (5) planning and regulation (see Fig 2). The first three were designed to capture the interpersonal dimension of ToM, as they focus on recognizing and inferring other people’s mental states, whereas the last two were designed to capture the intrapersonal dimension, as they focus on reflecting on and identifying one’s own mental states, as well as regulating responses in context.

To measure these dimensions, the initial focus was on the emotional states of happiness, sadness, anxiety, anger, frustration, calmness and relaxation. Different response formats were considered for each section, including a Likert-type scale with options such as always, almost always, sometimes and never, as well as a numerical scale from 1 to 5, where 1 represented totally disagree and 5 represented totally agree.

Based on this initial structure, the interpersonal dimension, consisted of the following sections of the questionnaire:

Emotion Recognition: This refers to the ability to identify emotional states. The questions were designed to explore how often participants recognized each emotional state in children with ASD.

Making Inferences: This refers to the ability to attribute causes to emotional states. It assesses how often participants identified the factors or situations that led to each emotional state.

Empathic Response: This refers to the ability to respond to emotional expressions. The questions explore how often participants knew how to respond to or understand children’s emotional expressions.

For the intrapersonal dimension, the sections were as follows:

Reflective Component: This refers to the internal reflective processes concerning one’s own thoughts and emotions and consisted of two subsections. The first subsection assesses how often participants identified their own thoughts and emotions when children with ASD exhibit the emotional states included in the questionnaire. The second subsection, related to reflective ability, explored how often participants reflected on their own thoughts and emotions.

Planning and Regulation: This refers to internal processes involved in anticipating responses and managing one’s own reactions in context and consisted of two subsections. The first, planning ability, assesses how often participants were able to plan interventions to promote or prevent each of the emotions included. The second, emotion regulation ability, examined how often participants were able to regulate their own emotions when children with ASD express any of the emotions studied.

In its original version, the instrument consisted of 84 items. Different question types and response formats were considered necessary for each section. Once the design and construction phases were completed, the questionnaire underwent expert validation through expert judgment and subsequent cognitive interviews with key informants. This process allowed the questionnaire to be refined before its administration in educational communities.

### Validation by expert judgment

Considering that there are no specialists in the evaluation of ToM-related skills in the Chilean context, and that this instrument was designed for education professionals working in school settings implementing inclusion-related policies, five experts in school inclusion were invited to participate. They represented different higher education institutions and school integration teams. Of these experts, two were professionals working in educational communities with responsibilities related to inclusion, and three were academics. Experts were identified through academic networks and publicly available information form Chilean universities.

For inclusion in this process, professional background and expertise, as reflected in publications and studies related to school inclusion and ASD, were considered to ensure the selection of professionals with substantial experience in this area. Based on individual credentials, the K coefficient was determined in accordance with the guidelines of Marín-González [66]. This process involved the application of two instruments. The first consisted of a self-assessment scale in which each expert assigned themselves a score between 1 and 10 according to their expertise in the field of study. This self-assessment yielded the first K coefficient. The second instrument consisted of a table in which each expert responded according to their experience and knowledge. Each response was assigned a specific value, and the sum of these values determined the second K coefficient. The first and second coefficients were averaged. To be considered experts, participants had to obtain an average score equal to or greater than 0.7. The experts consulted obtained average scores between 0.8 and 0.9; therefore, the validation instrument was sent to each of them by email.

### Cognitive interviews

A convenience sampling method was used for the cognitive interviews. Key informants were selected from school settings implementing inclusion-related policies. To recruit professionals working in these settings, authorization was first requested from the authorities of each educational institution. Once authorization was granted, members of the integration team were invited to participate. In addition, a school psychologist specializing in ASD and with extensive professional experience was contacted. Finally, two mothers of children diagnosed with ASD were invited to participate; they were contacted by telephone and agreed to participate voluntarily.

For the cognitive interviews, the guidelines proposed by Rodríguez and Smith-Castro and by Molina [67,68] were followed. These interviews were conducted in October 2022. Because health restrictions related to the Sars-CoV-2 pandemic were still in place at that time, all interviews were conducted via videoconference using the Zoom platform. A total of seven informants participated (see Table 1).

**Table 1:** Main characteristics of cognitive interview participants.

Part A: Parents or caregivers of children diagnosed with ASD					
	**Type of participant**	**Child’s age**	**Age at diagnosis**	**Years in school**	**Number of schools attended**
**Inter. 1**	Mother	9 years	3 ½ years	5	2
**Inter. 7**	Mother	13 years	6 years	8	2
**Part B: Education Professionals**					
**Participant**	**Profession**			**Years of work experience**	
**Inter. 2**	School psychologist specializing in ASD			20 years	
**Inter. 3**	Special education teacher			2 months	
**Inter. 4**	Special education teacher (SLI focus)*			2 years	
**Inter. 5**	Special education teacher			7 years	
**Inter. 6**	Speech therapist			4 years	

*SLI = Specific language impairment; Inter = Interviewee.

Before each interview began, participants were asked to explicitly confirm their voluntary participation, and an audiovisual record of their consent was kept. Each interview began with a greeting, an expression of gratitude for their participation, and a statement informing participants that the session would be audiovisually recorded. After this explanation, consent was obtained. All audiovisual recordings have been kept under the exclusive custody of the principal investigator and stored securely and confidentially in MP4 format. These records will be permanently destroyed one year after the publication of this article.

The cognitive interview began once the participants provided consent. First, the general instructions of the instrument were read aloud, and participants were asked whether the information was understandable or whether additional information should be included. For each item, participants were asked to indicate whether the question was easy to answer and whether the interpretation was clear. Because the questionnaire assessed each ToM-related skill using different grammatical structures and response formats, participants were asked which format was easiest to answer and most relevant to the school context. They were also asked whether the wording of any question made it uncomfortable to answer, either because it could bias responses toward one option or because of discomfort with the wording itself.

### Instrument refinement

Based on the contributions obtained through expert judgment and cognitive interviews, an initial refinement of the instrument was carried out. The refined version of the instrument was submitted for approval to the Ethics and Biosafety Committee of the University of Concepción.

### Pilot Study

After approval was obtained from the Ethics and Biosafety Committee, the pilot study was initiated. The refined instrument was administered to teachers and integration team professionals between June and December 2023. The inclusion criteria were being a teacher or a member of an integration team in educational institutions in Concepción Province, Chile, that had implemented the School Integration Program and enrolled students diagnosed with ASD.

At the time of the instrument’s administration, Concepción Province had 324 educational institutions that had implemented the PIE program. Of these, 281 (87.7%) reported enrolling students diagnosed with ASD. Initially, institutions were selected using simple random sampling. Once selected, principals were contacted by email or telephone to request a meeting and obtain authorization to enter the school community and administer the questionnaire. Of the 281 institutions, only 30 responded to the request, representing 10.68% of the total. Of these, nine explicitly stated that they did not intend to participate in the study, and two could not be included due to external circumstances, such as union mobilizations. As a result, the final sample consisted of 19 institutions, representing 6.79% of the total. Consequently, the sample was classified as a convenience sample. Because participation depended on the institutional authorization and voluntary uptake, the final sample was determined by availability and willingness to participate.

According to records from the Ministry of Education, the institutions included in this study employed a total of 629 education professionals. Of these, 291 participated in the study, representing 46.26% of the total. A minimum sample size of 240 participants was required to achieve a 5% margin of error, indicating that the final sample size was adequate for subsequent analyses. Participants had a mean age of 36.86 years, a mean work experience of 10.56 years, and a mean length of service at their institution, a factor associated with job stability, of 6.25 years.

Regarding the sociodemographic characteristics of the sample, it is important to acknowledge the presence of missing data, as some participants were unwilling to provide personal information. For age, 238 participants answered the question, resulting in 53 missing responses (18%). For years of work experience, 260 participants responded, resulting in 31 missing responses (11%). For length of service at the institution, 244 participants responded, resulting in 47 missing responses (16%).

The reasons for these levels of missing data are beyond the scope of this study; however, they may reflect reluctance or discomfort in providing personal information.

The instrument was administered in person. A meeting was requested with all teachers and integration team professionals. Once participants had gathered, they were provided with general information about the study, including an explicit statement that responses were anonymous, confidential, and voluntary. Professionals who agreed to complete the questionnaire were required to sign the corresponding informed consent form. During administration, which lasted approximately 30 minutes, the researcher did not intervene, allowing each participant to respond freely according to their own perceptions.

### Exploratory factor analysis

After the instrument had been administered, an exploratory factor analysis was performed using RStudio version 2023.12.1 and the lavaan package. Maximum likelihood extraction was used, and varimax rotation was applied to obtain an interpretable simple structure in this initial pilot phase, consistent with recommended practices for early-stage scale development and EFA reporting [69–71]. The choice of varimax rotation is also consistent with prior pilot and validation studies that have used varimax to examine and refine the latent structure of newly developed questionnaires [72–76]. Given the sample size of only 291 participants and the exploratory nature of this pilot study, a confirmatory factor analysis was not performed. At this stage, EFA was considered more appropriate because it allowed for an initial examination of the behavior of the variables and informed a second refinement of the instrument.

## Results

The objective of this study was to design, construct, and pilot test an instrument to measure ToM-related skills in education professionals who work with students diagnosed with ASD. This section presents the results of the initial refinement of the instrument, based on expert judgment and cognitive interviews, as well as the exploratory factor analysis conducted to examine its internal structure.

### Instrument refinement

The initial phase consisted of designing the instrument, which required identifying key ToM-related components relevant to teachers and integration team professionals. This was achieved through a comprehensive review of the literature and consultation with experts in the field. The instrument was intended to assess multiple ToM-related dimensions, and the design process resulted in an initial set of 84 items, which were subjected to expert judgment and cognitive interviews to support content validity.

The experts’ contributions were mainly related to the use of inclusive language in the Chilean context, such as differentiating between boys and girls and including the term “condition” (“condición” in Spanish) to refer to autism. This recommendation is consistent with national inclusion guidelines [77] which suggest referring to autism as a condition rather than as a disorder. This suggestion was adopted, and both terms, ASD and ASC, were incorporated into the instrument, as participants expressed divided views on how to refer to autism; most associated it more closely with a disorder (ASD) than with a condition (ASC). In addition, experts suggested reducing the number of questions to prevent the instrument from being excessively long or redundant.

Among the most relevant comments, Expert 1 noted that, in the section on empathic response, “people with ASC show different empathic responses”, and suggested changing the term “capacity” in the section on reflective capacity. Expert 2 referred to the repetitiveness and redundancy of the items. These comments were incorporated into the cognitive interviews, and participants’ responses were considered in the development of the final instrument.

During the cognitive interviews, participants suggested replacing the emotion “happy” (“feliz” in Spanish), with “joyful” (“alegre” in Spanish) as the former was perceived as more subjective and could therefore generate ambiguous responses or confusion when answering. The emotions “frustrated” and “angry” were also merged, as most respondents reported difficulty identifying them separately. The term “relaxed” was eliminated because it was considered an intrapersonal emotional state and therefore difficult to identify in children with ASD.

A paragraph was added to the general instructions of the instrument explicitly stating that the purpose of the research was not to evaluate the quality of teaching provided by teachers or integration team professionals, to avoid response bias. It was also clarified that this instrument was not intended to judge teaching practices. Regarding the response format, all items were revised to ensure that they used similar response options, and the options that performed best during the cognitive interviews were selected. These were: always, almost always, sometimes, and never.

Based on the comments provided during the expert judgment and cognitive interview processes, the instrument was refined by reducing the number of items, refining the wording, and modifying some concepts to facilitate understanding. The number of items was reduced from 84 in the original version to 35 in the refined version. Redundant items that respondents found difficult to answer were eliminated; response formats were also adjusted to better reflect respondents’ intended answers.

Thus, the refined version of the questionnaire consisted of the following sections: (1) Emotion recognition, comprising 5 items; (2) Making inferences, comprising 5 items; (3) Empathic response, comprising 5 items; (4) Reflective component, comprising 10 items, with the first five items assessing the identification of one’s own thoughts and emotions and the next five items assessing the frequency of reflection on one’s own thoughts and emotions, and [5] Planning and regulation, comprising 10 items, with the first five items assessing planning ability, and the next five items assessing the ability to regulate one’s own emotions (see Table 2).

**Table 2:** Distribution of items in the refined instrument.

Section Name	Number of items
Emotion Recognition	5
Making Inferences	5
Empathic Response	5
Reflective Component	10
Planning and emotion regulation ability	10
Total	35

Number of items = Total number of items included in each section.

The refined version of the instrument was submitted for approval to the Ethics and Biosafety Committee of the University of Concepción.

### Exploratory factor analysis

Following the design phase, the instrument was administered to a sample of 291 participants, consisting of teachers and integration team professionals. The pilot study aimed to evaluate the instrument’s reliability and preliminary validity. Descriptive statistics were calculated to summarize the demographic characteristics of the sample, including age, work experience, and length of service at the institution.

An Exploratory Factor Analysis (EFA) was conducted to examine the underlying structure of the instrument using maximum likelihood extraction and varimax rotation. Sampling adequacy was supported by a Kaiser-Meyer-Olkin (KMO) coefficient of 0.91, which is considered meritorious [78], and Bartlett’s test of sphericity indicated that the correlation matrix was suitable for factor analysis (*X*^*2*^ = 9233.139; df = 595; p = < 0.001). The number of factors to retain was determined using Horn’s parallel analysis [79] with 1000 and 5000 bootstrap samples, which suggested a five-factor solution. Items were retained on a given factor when their primary loading was ≥ 0.40 and no secondary loading reached 0.40 on any other factor, indicating the absence of salient cross-loadings.

The EFA yielded a five-factor solution with a simple structure: all items showed their highest loading on a single factor, and no salient cross-loadings were observed (see Table 3). Factor 1 comprised items capturing the interpersonal dimension, including emotion recognition, making inferences, and empathic response. Factor 2 included items assessing how frequently participants engage in a reflective processes about their own thoughts and emotions when interacting with children diagnosed with ASD. Factor 3 included items related to the ability to plan actions or activities aimed at promoting or preventing specific emotional states in these children. Factor 4 comprising items referring to the perceive ability to regulate one’s own emotions in response to children’s emotional expressions. Finally, Factor 5 included items assessing the ability to identify one’s own emotions and thoughts when faced with children’s emotional expressions.

**Table 3:** Exploratory factor analysis, theory of mind instrument, initial version.

ID		Factor loadings				
**RE1**	Do you recognize when the child with a diagnosis of ASD/ASC is joyful?	**0.46**	0.05	0.18	0.07	0.23
**RE2**	Do you recognize when the child with a diagnosis of ASD/ASC is sad?	**0.51**	0.00	0.27	0.08	0.20
**RE3**	Do you recognize when the child with a diagnosis of ASD/ASC is anxious?	**0.52**	0.16	0.09	0.10	0.15
**RE4**	Do you recognize when the child with a diagnosis of ASD/ASC is frustrated or angry?	**0.56**	0.09	0.15	0.22	0.11
**RE5**	Do you recognize when the child with a diagnosis of ASD/ASC is calm?	**0.50**	0.14	0.10	0.19	0.12
**RI1**	Do you recognize what situations or things allow the child with a diagnosis of ASD/ASC to feel joyful?	**0.72**	0.13	0.14	0.10	0.14
**RI2**	Do you recognize what situations or things allow the child with a diagnosis of ASD/ASC to feel sad?	**0.75**	0.10	0.10	0.11	0.07
**RI3**	Do you recognize what situations or things allow the child with a diagnosis of ASD/ASC to feel anxious?	**0.67**	0.14	0.05	0.13	0.05
**RI4**	Do you recognize what situations or things allow the child with a diagnosis of ASD/ASC to feel frustrated or angry?	**0.68**	0.13	0.12	0.08	0.14
**RI5**	Do you recognize what situations or things allow the child with a diagnosis of ASD/ASC to feel calm?	**0.70**	0.23	0.06	0.16	0.10
**Rem1**	I am clear about how I should act or what to do when the child with a diagnosis of ASD/ASC is joyful	**0.53**	0.19	0.20	0.14	0.19
**Rem2**	I am clear about how I should act or what to do when the child with a diagnosis of ASD/ASC is sad	**0.62**	0.11	0.28	0.15	0.19
**Rem3**	I am clear about how I should act or what to do when the child with a diagnosis of ASD/ASC is anxious	**0.60**	0.12	0.29	0.12	0.18
**Rem4**	I am clear about how I should act or what to do when the child with a diagnosis of ASD/ASC is frustrated or angry	**0.56**	0.08	0.25	0.12	0.12
**Rem5**	I am clear about how I should act or what to do when the child with a diagnosis of ASD/ASC is calm	**0.57**	0.24	0.24	0.11	0.18
**CR1**	I am aware of the thoughts and emotions that come to me when children with a diagnosis of ASD/ASC are joyful	0.31	0.27	0.14	0.12	**0.78**
**CR2**	I am aware of the thoughts and emotions that come to me when children with a diagnosis of ASD/ASC are sad	0.28	0.32	0.17	0.15	**0.79**
**CR3**	I am aware of the thoughts and emotions that come to me when children with a diagnosis of ASD/ASC are anxious	0.31	0.37	0.12	0.13	**0.62**
**CR4**	I am aware of the thoughts and emotions that come to me when children with a diagnosis of ASD/ASC are frustrated or angry	0.24	0.35	0.15	0.19	**0.58**
**CR5**	I am aware of the thoughts and emotions that come to me when children with a diagnosis of ASD/ASC are calm	0.30	0.38	0.06	0.11	**0.69**
**CR6**	I reflect on the thoughts and emotions that arise in me when children with a diagnosis of ASD/ASC are joyful	0.27	**0.75**	0.10	0.08	0.34
**CR7**	I reflect on the thoughts and emotions that come to me when children with a diagnosis of ASD/ASC are sad	0.19	**0.86**	0.17	0.17	0.25
**CR8**	I reflect on the thoughts and emotions that come to me when children with a diagnosis of ASD/ASC are anxious	0.19	**0.87**	0.17	0.19	0.23
**CR9**	I reflect on the thoughts and emotions that come to me when children with a diagnosis of ASD/ASC are frustrated or angry	0.17	**0.84**	0.15	0.16	0.18
**CR10**	I reflect on the thoughts and emotions that come to me when children with a diagnosis of ASD/ASC are calm	0.25	**0.73**	0.15	0.11	0.31
**PR1**	I am able to plan actions to make the child with a diagnosis of ASD/ASC feel joyful	0.28	0.21	**0.67**	0.21	0.23
**PR2**	I am able to plan actions to prevent the child with an ASD/ASC diagnosis from feeling sad	0.28	0.13	**0.82**	0.20	0.14
**PR3**	I am able to plan actions to prevent the child with an ASD/ASC diagnosis from feeling anxious	0.29	0.14	**0.80**	0.21	0.07
**PR4**	I am able to plan actions to prevent the child with an ASD/ASC diagnosis from feeling frustrated or angry	0.24	0.09	**0.75**	0.21	0.00
**PR5**	I am able to plan actions to make the child with a diagnosis of ASD/ASC feel calm	0.27	0.20	**0.61**	0.21	0.16
**PR6**	I am able to regulate my own emotions when the child with a diagnosis of ASD/ASC is joyful	0.17	0.13	0.31	**0.62**	0.12
**PR7**	I am able to regulate my own emotions when the child with a diagnosis of ASD/ASC is sad	0.20	0.08	0.21	**0.80**	0.21
**PR8**	I am able to regulate my own emotions when the child with a diagnosis of ASD/ASC is anxious	0.25	0.11	0.15	**0.88**	0.09
**PR9**	I am able to regulate my own emotions when the child with a diagnosis of ASD/ASC is frustrated or angry	0.23	0.12	0.08	**0.79**	0.07
**PR10**	I am able to regulate my own emotions when the child with a diagnosis of ASD/ASC is calm	0.15	0.21	0.23	**0.66**	0.11
	**% variance explained**	**19%**	**13%**	**10%**	**10%**	**9%**

Considering these results, with factors explaining between 9% and 19% of the variance, the five-factor solution was retained. Given that the first factor grouped items from the interpersonal dimension, explained 19% of the variance, and showed strong item loadings, a second factor analysis was conducted using only the items within this factor. Horn’s parallel analysis suggested a three-factor solution. The factor loading pattern distinguished three subdomains: emotion recognition, making inferences, and empathic response (see Table 4). Accordingly, the first factor was further specified as comprising three subfactors aligned with these interpersonal ToM-related skills.

**Table 4:** Exploratory factor analysis, theory of mind instrument, interpersonal component.

ID		Factor loadings		
**RE1**	Do you recognize when the child with a diagnosis of ASD/ASC is joyful?	0.12	0.14	**0.82**
**RE2**	Do you recognize when the child with a diagnosis of ASD/ASC is sad?	0.21	0.19	**0.75**
**RE3**	Do you recognize when the child with a diagnosis of ASD/ASC is anxious?	0.19	0.36	**0.46**
**RE4**	Do you recognize when the child with a diagnosis of ASD/ASC is frustrated or angry?	0.23	0.36	**0.54**
**RE5**	Do you recognize when the child with a diagnosis of ASD/ASC is calm?	0.24	0.21	**0.62**
**RI1**	Do you recognize what situations or things allow the child with a diagnosis of ASD/ASC to feel joyful?	0.30	**0.63**	0.33
**RI2**	Do you recognize what situations or things allow the child with a diagnosis of ASD/ASC to feel sad?	0.30	**0.66**	0.30
**RI3**	Do you recognize what situations or things allow the child with a diagnosis of ASD/ASC to feel anxious?	0.22	**0.79**	0.10
**RI4**	Do you recognize what situations or things allow the child with a diagnosis of ASD/ASC to feel frustrated or angry?	0.22	**0.74**	0.24
**RI5**	Do you recognize what situations or things allow the child with a diagnosis of ASD/ASC to feel calm?	0.30	**0.63**	0.32
**Rem1**	I am clear about how I should act or what to do when the child with a diagnosis of ASD/ASC is joyful	**0.78**	0.17	0.20
**Rem2**	I am clear about how I should act or what to do when the child with a diagnosis of ASD/ASC is sad	**0.79**	0.26	0.22
**Rem3**	I am clear about how I should act or what to do when the child with a diagnosis of ASD/ASC is anxious	**0.73**	0.32	0.15
**Rem4**	I am clear about how I should act or what to do when the child with a diagnosis of ASD/ASC is frustrated or angry	**0.62**	0.30	0.16
**Rem5**	I am clear about how I should act or what to do when the child with a diagnosis of ASD/ASC is calm	**0.77**	0.19	0.28
	**% variance explained**	**22%**	**20%**	**18%**

Based on these results, and considering the literature on ToM and the similar proportions of variance explained across factors, the instrument was organized as follows: (1) Section 1 comprises Factor 1 and covers the interpersonal ToM-related skills (emotion recognition, making inferences, and empathic response); this section is referred to as “identification of others’ mental states”. (2) Section 2 comprises Factors 2, 3, 4, and 5, and focuses on intrapersonal ToM-related skills; this section is referred to as “identification of one’s own mental states” (see Table 5).

**Table 5:** Streamlined version of the instrument.

ID		Factor loadings				
	**Section 1: Identification of others’ mental states**					
	**1.1: Emotions recognition**					
RE1	Do you recognize when the child with a diagnosis of ASD/ASC is joyful?	**0.46**				
RE2	Do you recognize when the child with a diagnosis of ASD/ASC is sad?	**0.51**				
RE3	Do you recognize when the child with a diagnosis of ASD/ASC is anxious?	**0.52**				
RE4	Do you recognize when the child with a diagnosis of ASD/ASC is frustrated or angry?	**0.56**				
RE5	Do you recognize when the child with a diagnosis of ASD/ASC is calm?	**0.50**				
	**1.2: Making inferences**					
RI1	Do you recognize what situations or things allow the child with a diagnosis of ASD/ASC to feel joyful?	**0.72**				
RI2	Do you recognize what situations or things allow the child with a diagnosis of ASD/ASC to feel sad?	**0.75**				
RI3	Do you recognize what situations or things allow the child with a diagnosis of ASD/ASC to feel anxious?	**0.67**				
RI4	Do you recognize what situations or things allow the child with a diagnosis of ASD/ASC to feel frustrated or angry?	**0.68**				
RI5	Do you recognize what situations or things allow the child with a diagnosis of ASD/ASC to feel calm?	**0.70**				
	**1.3: Empathic response**					
Rem1	I am clear about how I should act or what to do when the child with a diagnosis of ASD/ASC is joyful	**0.53**				
Rem2	I am clear about how I should act or what to do when the child with a diagnosis of ASD/ASC is sad	**0.62**				
Rem3	I am clear about how I should act or what to do when the child with a diagnosis of ASD/ASC is anxious	**0.60**				
Rem4	I am clear about how I should act or what to do when the child with a diagnosis of ASD/ASC is frustrated or angry	**0.56**				
Rem5	I am clear about how I should act or what to do when the child with a diagnosis of ASD/ASC is calm	**0.57**				
	**Section 2: Identification of one’s own mental states**					
	**Ability to identify one’s own thoughts and emotions**					
CR1	I am aware of the thoughts and emotions that come to me when children with a diagnosis of ASD/ASC are joyful					**0.78**
CR2	I am aware of the thoughts and emotions that come to me when children with a diagnosis of ASD/ASC are sad					**0.79**
CR3	I am aware of the thoughts and emotions that come to me when children with a diagnosis of ASD/ASC are anxious					**0.62**
CR4	I am aware of the thoughts and emotions that come to me when children with a diagnosis of ASD/ASC are frustrated or angry					**0.58**
CR5	I am aware of the thoughts and emotions that come to me when children with a diagnosis of ASD/ASC are calm					**0.73**
	**Reflective ability**					
CR6	I reflect on the thoughts and emotions that arise in me when children with a diagnosis of ASD/ASC are joyful		**0.75**			
CR7	I reflect on the thoughts and emotions that come to me when children with a diagnosis of ASD/ASC are sad		**0.86**			
CR8	I reflect on the thoughts and emotions that come to me when children with a diagnosis of ASD/ASC are anxious		**0.87**			
CR9	I reflect on the thoughts and emotions that come to me when children with a diagnosis of ASD/ASC are frustrated or angry		**0.84**			
CR10	I reflect on the thoughts and emotions that come to me when children with a diagnosis of ASD/ASC are calm		**0.73**			
	**Planning ability**					
PR1	I am able to plan actions to make the child with a diagnosis of ASD/ASC feel joyful			**0.67**		
PR2	I am able to plan actions to prevent the child with an ASD/ASC diagnosis from feeling sad			**0.82**		
PR3	I am able to plan actions to prevent the child with an ASD/ASC diagnosis from feeling anxious			**0.80**		
PR4	I am able to plan actions to prevent the child with an ASD/ASC diagnosis from feeling frustrated or angry			**0.75**		
PR5	I am able to plan actions to make the child with a diagnosis of ASD/ASC feel calm			**0.61**		
	**Emotion regulation ability**					
PR6	I am able to regulate my own emotions when the child with a diagnosis of ASD/ASC is joyful				**0.62**	
PR7	I am able to regulate my own emotions when the child with a diagnosis of ASD/ASC is sad				**0.80**	
PR8	I am able to regulate my own emotions when the child with a diagnosis of ASD/ASC is anxious				**0.88**	
PR9	I am able to regulate my own emotions when the child with a diagnosis of ASD/ASC is frustrated or angry				**0.79**	
PR10	I am able to regulate my own emotions when the child with a diagnosis of ASD/ASC is calm				**0.66**	
	**% variance explained**	**19%**	**13%**	**10%**	**10%**	**9%**

The reliability analysis showed Cronbach’s alpha coefficients above 0.80 for all factors (see Table 6), providing promising evidence to support further application of the instrument in other educational communities. However, these findings should be interpreted cautiously, as they cannot be generalized without replication in larger and more diverse samples across educational communities in different locations.

**Table 6:** Reliability analysis of the Theory of Mind questionnaire.

		N	*α*	Number of items included
**Factor 1**	**Identification of others’ mental states**	285	0.92	15
	Factor 1.1: Emotions recognition	289	0.84	5
	Factor 1.2: Making inferences	288	0.89	5
	Factor 1.3: Empathic response	288	0.90	5
	**Identification of one’s own mental states**			
**Factor 2**	Reflective ability	291	0.96	5
**Factor 3**	Planning ability	287	0.92	5
**Factor 4**	Emotion regulation ability	289	0.91	5
**Factor 5**	Ability to identify one’s own thoughts and emotions	289	0.93	5

n = number of participants who answered the items associated with each factor.

## Discussion and conclusions

This article presents the development of a self-report instrument to assess ToM-related skills in education professionals who work with students diagnosed with ASD in school settings implementing inclusion-related policies. Within the framework of a broader project examining educators’ ToM-related skills in relation to school processes, this article focuses specifically on the design and exploratory psychometric evaluation of the instrument’s first pilot study. These preliminary results provide an initial description of how interpersonal and intrapersonal ToM-related dimensions are organized in a sample of teachers and integration team professionals from schools in the Province of Concepción, Chile.

To our knowledge, this is one of the first studies to examine the factor structure of ToM-related self-report measures specifically in education professionals working in school settings implementing inclusion-related policies, and to empirically differentiate between an integrated interpersonal dimension and more differentiated intrapersonal components.

ToM, understood as the capacity to attribute mental states to oneself and others [1–4], has been linked to socio-emotional processes that are fundamental in the classroom [80,81], such as the development of socio-cognitive skills, emotion regulation, and mental health [20]. In educational psychology, these processes are integrated into broader frameworks of teacher cognition, socio-cognitive competence, and mentalization in teacher–student relationships, in which teachers’ interpretations of their own and others’ mental states may shape classroom climate, relationship quality, and opportunities for participation [24,82,83]. These ToM-related processes are also relevant when considering Ainscow’s view that progress toward inclusive education requires a cultural change in how schools understand and respond to student diversity, including recognition of students’ individual experiences and emotional worlds [84,85].

Most empirical work on ToM has focused on child samples, neuropsychiatric disorders, or experimental tasks [26], and existing instruments often assess specific ToM skills but are insufficient for identifying how ToM is used in school contexts implementing inclusion-related policies. The present study addresses this gap by proposing an instrument centered on teachers and professionals working in educational settings, constructed around a theoretically derived profile of interpersonal and intrapersonal ToM-related skills relevant to interactions with autistic students in schools implementing inclusion-related policies.

The instrument was initially constructed around five components aligned with interpersonal and intrapersonal dimensions, with 35 items framed around emotional states frequently observed in interactions with students diagnosed with ASD. The exploratory factor analysis did not fully reproduce this structure. Instead, the analysis distributed the 35 items across five factors, which were grouped into two broad sections: (1) identification of others’ mental states (interpersonal dimension) and (2) identification of one’s own mental states (intrapersonal dimension).

The interpersonal dimension emerged as a single factor encompassing items related to emotion recognition, making inferences, and empathic response. The intrapersonal dimension was represented by four factors reflecting the identification of one’s own thoughts and emotions, reflective processes, planning of actions, and regulation of one’s own emotions in interactions with autistic children. These results provide preliminary evidence of a two-domain structure that combines an integrated interpersonal factor with a more differentiated intrapersonal configuration. All factors showed high internal consistency, and the instrument appeared feasible to administer in school settings, with no substantial comprehension difficulties reported.

The interpersonal dimension was originally conceptualized as comprising three distinct components: emotion recognition, making inferences and empathic response. However, the exploratory factor analysis indicated that the items formed a single factor in this sample. A plausible interpretation is that, in everyday classroom practice, these interpersonal skills tend to be deployed simultaneously rather than sequentially or independently. When a student, particularly an autistic student, shows an emotional reaction, teachers are often required to notice the emotion, infer its possible causes, such as sensory overload, task difficulty or peer conflict, and decide how to respond empathically, all within a short period of time and under considerable situational demands. In this context, it may be more realistic to conceptualize interpersonal ToM-related skills as a tightly integrated socio-cognitive resource rather than as three clearly separable abilities.

This interpretation is consistent with studies on teachers’ mentalization and perspective-taking, which describe interpersonal socio-cognitive skills as highly interconnected in practice [24,82]. Research conducted in real classrooms suggests that teachers’ mentalization often involves the simultaneous integration of cognitive perspective-taking and sensitivity to students’ affective states when managing complex interactions, rather than the isolated use of discrete processes [19,21,80,86]. The present findings therefore converge with this literature: recognition of students’ emotions, attribution of causes, and empathic responding clustered into a single interpersonal factor when teachers and integration team professionals inferred mental states in schoolchildren diagnosed with ASD.

This unifactorial structure of the interpersonal dimension differs from more modular conceptualizations of ToM that treat emotion recognition, inference, and empathy as distinct components [10,11]. The current results suggest that these distinctions, although theoretically important, may not be directly reflected in how teachers and integration team professionals experience and report their everyday socio-cognitive work in classrooms implementing inclusion-related policies. This divergence should not be interpreted as a refutation of traditional models, but rather as an indication that, in applied educational contexts and at the self-report level, interpersonal ToM-related skills may manifest as a more integrated construct than is usually assumed. Given the characteristics of the sample analyzed in this study, this interpretation should be viewed with caution, as these are preliminary findings that require replication.

By contrast, the section covering the intrapersonal dimension, termed “identification of one’s own mental states”, showed a more differentiated pattern than initially expected. Instead of two broader components, namely the reflective component and the ability to plan and regulate emotions, the exploratory factor analysis identified four factors: (1) identification of one’s own thoughts and emotions, (2) reflective ability, (3) planning ability and (4) emotion regulation ability. This configuration only partially aligns with the initial theoretical model but is compatible with theories that describe intrapersonal ToM as multifaceted construct encompassing partially separable functions such as emotional awareness, self-reflection, anticipatory planning, and regulatory capacity [87].

These findings can be interpreted considering teachers lived experiences in classrooms implementing inclusion-related policies. Working with autistic students may require educators to manage their own emotions in challenging situations, such as episodes of frustration, anger, or anxiety. Reflective processes about these experiences may be postponed to another moment to prioritize anticipating how to act in similar situations in the future and regulating emotions in the moment to maintain a conducive learning environment. It is plausible that teachers experience and report these skills as distinct yet related demands. The four-factor structure for the identification of one’s own mental states observed here is consistent with this interpretation and suggests that teachers and integration team professionals differentiate several intrapersonal skills when interacting with autistic students.

From a theoretical standpoint, these findings are consistent with the ToM literature regarding the complexity of intrapersonal socio-cognitive skills, which distinguishes between awareness of internal states, reflective elaboration, and regulation [87,88]. These preliminary findings may contribute to extending this perspective to an applied educational context by providing factor-analytic evidence that teachers and integration teams professionals working in schools implementing inclusion-related policies, report differentiated intrapersonal ToM-related components when responding to situations involving autistic students. However, the data do not allow conclusions to be drawn about the mechanisms underlying these differences or about their practical implications for teaching or inclusion.

The combination of a unifactorial interpersonal structure and a multifactorial intrapersonal configuration is a central contribution of this study. In this context, the instrument and its preliminary structure offer a new perspective in three respects.

First, it questions the implicit assumption, present in some theoretical models, that the interpersonal components of ToM will emerge as clearly separable dimensions in applied settings [10,89,90]. The current preliminary findings suggest that, in the context of school implementing inclusion-related policies, interpersonal ToM-related skills, as reported by participants, may function as a coherent self-perceived socio-cognitive competence.

Second, it provides empirical support, in an educational sample, for considering the skills that comprise the intrapersonal ToM dimension as a multifaceted construct, in line with broader mentalization frameworks [91], but with a more detailed structure (identification of thoughts and emotions, reflective process, planning ability, and emotion regulation) than is typically operationalized in teacher research [11].

Third, it addresses a specific gap in the literature: the lack of instruments designed for education professionals, grounded in ToM, and explicitly oriented toward working with students diagnosed with ASD. Although existing mentalization scales provide valuable information, they are aimed at other populations or focus on broader constructs that are not tailored to the demands of classrooms implementing inclusion-related policies. The present study does not seek to replace these instruments but to complement them by offering a context-specific tool that captures interpersonal and intrapersonal ToM-related skills in educators.

However, the relevance of these results and their potential contributions must be interpreted cautiously. The present findings are exploratory, based on a single sample and a single analytic method, and have not yet been tested using confirmatory factor models. The main contribution of this work is to provide an empirically grounded and theoretically informed starting point for future research on ToM-related skills in teachers and integration team professionals.

Although this study did not assess classroom processes, pedagogical practices, or student outcomes, the findings have several conceptual implications for future research in school implementing inclusion-related policies. The interpersonal dimension, as an integrated factor, suggests that, in teachers’ self-reports, skills such as recognizing students’ emotions, inferring their causes, and responding empathically tend to form a single competence. Future studies could explore whether higher scores on this factor are associated with observable differences in classroom interactions, such as greater perceived emotional support, more sensitive responses to signals from children diagnosed with ASD, or more flexible adjustments to their needs.

Similarly, the intrapersonal dimension suggests possible avenues for understanding how educators’ internal processes relate to their professional performance in schools implementing inclusion-related policies. For example, future research could examine whether teachers who report greater emotional awareness and reflective ability also show different patterns of classroom management, different levels of burnout, or different perceptions of self-efficacy when working with students diagnosed with ASD. Other studies could explore whether planning and emotion regulation abilities are related to how teachers manage specific challenging situations, such as frustration or anxiety, emotions that may be associated with sensory overload, or to how they coordinate with integration teams and families.

These implications remain hypothetical at this stage and must be tested empirically. The present study does not support claims about the impact of ToM-related skills on classroom dynamics or the development of inclusive environments. Rather, it establishes a conceptual and measurement framework that can be used in future research to investigate these hypotheses.

Several limitations constrain the interpretation of the findings and indicate specific directions for future research.

First, expert participation in the content validation stage was limited in both number and diversity, and no specialists in ToM or psychometric methodology were included. Quantitative item-level ratings such as Aiken’s V or Lawshe’s coefficient were not collected due to the original length of the instrument. These limitations likely reduced the depth and breadth of conceptual and technical feedback and may have contributed to discrepancies between the initial theoretical structure and the empirical solution. Future research should address this limitation by incorporating a broader panel that includes experts in ToM, inclusive education, and psychometrics, and by applying formal content validity indices to support item selection and refinement.

Second, the sample was limited to a small number of institutions within a single Chilean province, with variable participation rates across schools. This limits the generalizability of the findings and raises questions about the stability of the factor structure. To address this limitation, future studies should implement stratified designs covering several regions of the country, or even adopt a transnational approach, involving diverse types of schools, educational levels, and professional roles. Stratified sampling could help obtain more representative samples and reduce selection bias.

Third, the present study relied exclusively on exploratory factor analysis. The proposed two-domain, five-factor structure remains provisional and may not be the only or the best representation of the data. Future research should conduct confirmatory factor analyses (CFA) to test the fit of this model against plausible alternatives, such as different factor solutions, higher-order structures, or bifactor models, and should examine measurement invariance across subgroups, for example, teachers versus integration team professionals. Longitudinal designs could also be used to assess the stability of factor scores over time.

Fourth, a substantial proportion of missing data in sociodemographic variables reduced the completeness of the sample description and may have introduced unknown biases. The present design did not allow us to investigate the reasons for nonresponse. Future studies should monitor missing data more systematically, explore whether nonresponse is associated with specific participant characteristics or school contexts, and consider strategies to reduce missing information.

Fifth, despite explicitly stating that responses were anonymous, confidential, and not intended to evaluate teaching practices, the instrument is based on self-report and may therefore be influenced by social desirability and self-presentation, particularly in professional contexts where respondents may feel evaluated. This common-method limitation may inflate associations among items and should be addressed in future studies by incorporating measures of social desirability. Therefore, scores should not be interpreted as direct indicator of participants’ actual ToM ability, but as a self-reported perceptions of ToM-related skills in applied educational contexts.

In summary, this study provides preliminary evidence of the internal structure and reliability of a new self-report instrument that assesses ToM-related skills in education professionals working with students diagnosed with ASD in school settings implementing inclusion-related policies. The findings suggest an integrated interpersonal dimension and a more differentiated intrapersonal configuration, offering a nuanced and empirically grounded view of how teachers and integration team professionals report ToM-related skills in an applied educational context.

Although constrained by important methodological limitations and its exploratory design, the study contributes to reaffirming the relevance of ToM for teachers’ work in classrooms implementing inclusion-related policies and identifies a specific gap in the literature: the need for teacher-centered, theory-based tools to assess socio-cognitive skills in relation to autism and inclusion. The proposed instrument is not a final product but a starting point for further refinement and validation. Future research building on this foundation may help clarify how interpersonal and intrapersonal ToM-related dimensions are structured in educators, how they relate to classroom processes, and how they might eventually be supported in ways that contribute to more inclusive and responsive educational environments.

## Supporting information

S1 File Self-report survey to assess the use of Theory of Mind Skills. (DOCX)
