# Supplementary material for: Design, development, and pilot study of a scale for educational professionals to assess theory of mind-related skills regarding children with ASD
Source: PLoS One. 2026 Aug 28;21(8):e0355044. doi: 10.1371/journal.pone.0355044 (PMC13524288; doi:10.1371/journal.pone.0355044)
Supplement: Supplementary file 1 [file pone.0355044.s001.docx]

**Self-report survey to assess the use of Theory of Mind skills.**

I. **Identification**

| Name of the establishment: | | |  | | | | | | | |  |
| --- | --- | --- | --- | --- | --- | --- | --- | --- | --- | --- | --- |
|  | | | *(write your answer here)* | | | | | | | | |
|  | | |  | | | | | | | | |
| Dependency |  | Municipal | | Years of work exp. | |  | | | | | |
| *(mark with an X))* |  | P. Subsidized | |  | | *(Write your answer here)* | | | | | |
|  |  | P. paid | | Years in this estab. | |  | | | | | |
|  |  | LES* | |  | | *(Write your answer here)* | | | | | |
|  |  |  | |  | |  | | | | | |
| Occupation: |  | | | | | | | | |  | |
|  | *(Write your answer here)* | | | | | | | | | | |
| Cur. Position: |  | | | | Age: |  | | *Years old* | | | |
|  | *(Write your answer here)* | | | |  | *(Write your answer here)* | | | | | |
|  |  |  | |  | |  | |  | | | |
| Have you ever had children with a diagnosis of ASD/ASC in your care? | | | | | |  | Yes |  | No | | |
|  | | | | | | *(mark with an X)* | | | | | |
|  | | | | | |  | | | | | |
| Currently caring for children with ASD/ASC diagnosis? | | | | | |  | Yes |  | No | | |
|  | | | | | | *(mark with an X)* | | | | | |
|  | | | | | |  | | | | | |
| Has received formal training to working with children with an ASD/ASC diagnosis? | | | | | |  | Yes |  | No | | |
|  | | | | | | *(marK with an X)* | | | | | |
|  | | | | | |  | | | | | |
| Currently, how many children with an ASD/ASC diagnosis are you caring for? | | | | | |  | | | | | |
|  | | | | | | *(Write your answer here)* | | | | | |

*LES = Local Education Service

**Section 1: Identification others’ mind mental states**

1.1 **Emotions recognition**

Based on your work experience, please answer by markig with an “X” the option that most represents you in relation to the following statements:

|  | | **Always** | **Almost always** | **Sometimes** | **Never** |
| --- | --- | --- | --- | --- | --- |
| 1. | Do you recognize when the child with a diagnosis of ASD/ASC is joyful? |  |  |  |  |
| 2. | Do you recognize when the child with a diagnosis of ASD/ASC is sad? |  |  |  |  |
| 3. | Do you recognize When the child with a diagnosis of ASD(ASC is anxious? |  |  |  |  |
| 4. | Do you recognize when the child with a diagnosis of ASD/ASC is frustrated or angry? |  |  |  |  |
| 5. | Do you recognize when the chil with a diagnosis of ASD/ASC is calm? |  |  |  |  |

1.2 **Making inference**

Based on your work experience, please answer by marking with an “X” the option that most represents you in relation to the following statements:

|  |  | **Always** | **Almost always** | **Sometimes** | **Never** |
| --- | --- | --- | --- | --- | --- |
| 6. | Do you recognize what situations or things allow the child with a diagnosis of ASD/ASC to feel joyful? |  |  |  |  |
| 7. | Do you recognize what situations or things allow the child with a diagnosis of ASD/ASC to feel sad? |  |  |  |  |
| 8. | Do you recognize what situations or things allow the child with a diagnosis of ASD/ASC to feel anxiety? |  |  |  |  |
| 9. | Do you recognize what situations or things allow the child with a diagnosis of ASD/ASC to feel anger or frustration? |  |  |  |  |
| 10. | Do you recognize what situations or things allow the child with a diagnosis of ASD/ASC to feel calm? |  |  |  |  |

1.3 **Empathic response**

Based on your work experience, please answer by marking with an 2X” the option that most represents you in relation to the following statements:

|  |  | **Always** | **Almost always** | **Sometimes** | **Never** |
| --- | --- | --- | --- | --- | --- |
| 11. | I am clear about how I should act or what to do when the child with a diagnosis of ASD/ASC is joyful |  |  |  |  |
| 12. | I am clear about how I should act or what to do when the child with a diagnosis of ASD/ASC is sad |  |  |  |  |
| 13. | I am clear about how I should act or what to do when the child with a diagnosis of ASD/ASC is anxious |  |  |  |  |
| 14. | I am clear about how I should act or what to do when the child with a diagnosis of ASD/ASC is angry or frustrated |  |  |  |  |
| 15. | I am clear about how I should act or what to do when the child with a diagnosis of ASD/ASC is calm |  |  |  |  |

**Section 2: Identification one own’s mental states**

2.1 **Ability to identify one’s own thoughts and emotions**

Based on your work experience, please answer by marking with an “X” the option that most represents you in relation to the following statements:

|  |  | **Always** | **Almost always** | **Sometimes** | **Never** |
| --- | --- | --- | --- | --- | --- |
| 16. | I am aware of the thoughts and emotions that comes to me when the children with a diagnosis of ASD/ASC are joyful |  |  |  |  |
| 17. | I am aware of the thoughts and emotions that comes to me when the children with a diagnosis of ASD/ASC are sad |  |  |  |  |
| 18. | I am aware of the thoughts and emotions that comes to me when the children with a diagnosis of ASD/ASC are anxious |  |  |  |  |
| 19. | I am aware of the thoughts and emotions that comes to me when the children with a diagnosis of ASD/ASC are frustrated or angry |  |  |  |  |
| 20. | I am aware of the thoughts and emotions that comes to me when the children with a diagnosis of ASD/ASC are calm |  |  |  |  |

2.2 **Reflective ability**

Based on your work experience, please answer by marking with an “X” the option that most represents you in relation to the following statements:

|  |  | **Always** | **Almost always** | **Sometimes** | **Never** |
| --- | --- | --- | --- | --- | --- |
| 21. | I reflect on the thoughts and emotions that arise in me when children with a diagnosis of ASD/ASC are joyful |  |  |  |  |
| 22. | I reflect on the thoughts and emotions that arise in me when children with a diagnosis of ASD/ASC are sad |  |  |  |  |
| 23. | I reflect on the thoughts and emotions that arise in me when children with a diagnosis of ASD/ASC are anxious |  |  |  |  |
| 24. | I reflect on the thoughts and emotions that arise in me when children with a diagnosis of ASD/ASC are frustrated or angry |  |  |  |  |
| 25. | I reflect on the thoughts and emotions that arise in me when children with a diagnosis of ASD/ASC are calm |  |  |  |  |

2.3 **Planning ability**

Based on your work experience, please answer by marking with an “X” the option that most represents you in relation to the following statements:

|  |  | **Always** | **Almost always** | **Sometimes** | **Never** |
| --- | --- | --- | --- | --- | --- |
| 26. | I am able to plan actions to make the child with a diagnosis of ASD/ASC feel joyful |  |  |  |  |
| 27. | I am able to plan actions to prevent the child with an ASD/ASC diagnosis from feeling sad |  |  |  |  |
| 28. | I am able to plan actions to prevent the child with an ASD/ASC diagnosis from feeling anxious |  |  |  |  |
| 29. | I am able to plan actions to prevent the child with an ASD/ASC diagnosis from feeling frustrated or angry |  |  |  |  |
| 30. | I am able to plan actions to make the child with a diagnosis of ASD/ASC feel calm |  |  |  |  |

2.4 **Emotions regulation ability**

Based on your work experience, please answer by marking with an “X” the option that most represents you in relation to the following statements:

|  |  | **Always** | **Almost always** | **Sometimes** | **Never** |
| --- | --- | --- | --- | --- | --- |
| 31. | I am able to regulate my own emotions when the child with a diagnosis of ASD/ASC is joyful |  |  |  |  |
| 32. | I am able to regulate my own emotions when the child with a diagnosis of ASD/ASC is sad |  |  |  |  |
| 33. | I am able to regulate my own emotions when the child with a diagnosis of ASD/ASC is anxious |  |  |  |  |
| 34. | I am able to regulate my own emotions when the child with a diagnosis of ASD/ASC is frustrated or angry |  |  |  |  |
| 35. | I am able to regulate my own emotions when the child with a diagnosis of ASD/ASC is calm |  |  |  |  |
