# Supplementary material for: Design, development, and pilot study of a scale for educational professionals to assess theory of mind-related skills regarding children with ASD
Source: PLoS One. 2026 Aug 28;21(8):e0355044. doi: 10.1371/journal.pone.0355044 (PMC13524288; doi:10.1371/journal.pone.0355044)
Supplement: Supplementary file 2 [file pone.0355044.s003.docx]

**July 14, 2025**

**Editor**

**Plos One**

**Of our consideration:**

We are submitting the revised manuscript entitled **“Desingn, construction and pilot study of an instrument to evaluate theory of mind skills in education professionals”,** to consider its publication as a Research Article in Plos One.

The comments made by the review team have been addressed and the corresponding modifications are detailed below:

1. Abstract

- **Reviewer 1:** “The abstract begins to discussing children with ASD, but the main focus of the manuscript is an instrument, not a study involving children. Lines 32-33: there is a lack of alignment between the title (wich refers to professionals) and the objective stated in the abstract (which refers to children). Lines 34-36: A second objective is introduced: “to present the desing, construction, and validation of an instrument that assesses the use of Theory of Mind skills in educational settings, along with the results from its first pilot study. This is unclear. Is the instrument intended for use with children or with teachers in educational settings? Furthermore, why do the authors refer to ‘validation’ if this is only a pilot study? The number of items included in the instrument is not mentioned. Also, a correlation of .37 is relatively weak and should be interpreted cautiously. Additionally, it is unclear why correlations between items are reported. The results of the exploratory factor analysis are not mentioned in the abstract, despite being crucial for establishing the internal structure of the instrument”
- **Reviewer 2:** First, the abstract would benefit from greater terminological specific. The phrase ‘use of the theory of mind skills’ is ambiguous: it is unclear whether the instrument assesses children’s ability to apply Theory of Mind (ToM) in educational contexts or teachers’ perceptions of those skills. Similary, the statement that instrument ‘assesses the use of theory of mind skills in educational settings’ does not specify whether the subjects of assessment are teachers or children with ToM related difficulties. I recommend explicity identifying the target population. Finally, the abastract inconsistently mixes present and past tense (e.g., ‘presents’ vs. ‘was administered’); a uniform past-tense narrative would align better with standard scientific conventions”

In response to these comments, the abstract of the article is completely modified, focusing in the construction of the instrument. The results of the exploratory factor analysis, the number of sections and resulting items are included. The title of the article is modified, and the word “validation” is eliminated, following the suggestion of Reviewer 1.

2. Introduction

- **Reviewer 1:** “The first pages of the introduction address a wide range of Theory of Mind (ToM) topics: definitions, development, neurological correlates, classifications, ASD, and ToM deficits in ASD. However, these topics are not directly related to the stated objective of the manuscript. The introduction should be rewritten to provide a focused rationale for the construction of the instrument. Additionally, there is a shift in focus: the manuscript discusses children with ASD and educational communities, but the emphasis should be in teachers, as suggested by the title”
- **Reviewer 2:** “In the introduction, I suggest a comprehensive language review to improve readability. In particular, the sentence beginning ‘An implicit one… (line 76) is structurally convoluted and suffers from unclear subject-verb agreement; Splitting it into two sentences and clearly defining each clause´s subject would significantly aid comprehension. Additionally, several passages (e.g.. line 136) are overly long and could be divided into shorter more focused sentences. There is also a formatting issue between lines 211 and 212, where an opening quotation mark is not closed, and a period is missing at the end of the sentence on line 239; please correct these errors”.

The suggestions made by the review team are accepted. The introduction has been modified to focus on the objective of the article. The theoretical foundations of the theory of mind construct and its multidimensionality (cognitive, affective, intra and interpersonal) are maintained. The lack of previous instruments assessing how ToM skills are used in social interactions without neuropsychiatric deficits is described. Writing is also modified to justify its importance in the context of inclusive education professionals. The grammatical errors in lines 211, 212 and 239 suggested by reviewer 2 are also corrected.

3. Methods and results

- **Reviewer 1:** Method: “The method section should follow a clear structure with subsections such as Participants, Instrument, Procedure, and Data Analysis. Currently, the narrative style makes the content difficult to follow. The exploratory factor analysis should include all items, not just dimensions. The authors should choose one reliability index -either Crombach’s aloha or McDonald’s omega – and justify their choice. The raw data are not available in public repository. Do the authors have any concerns or limitations regarding data sharing?”

Results: “The presentation of the results should be improved by clearly distinguishing the different stages of the instrument’s development. This could be achieved through subheadings and brief descriptions of each phase. Greater emphasis should be placed on the application of the instrument to the sample of 291 participants. The figures are not viewable; only a dark square appears. Thos must be corrected to allow proper evaluation of the data”.

- **Reviewer 2**: To improve the manuscript’s organization, I recommend dividing the Methods and Results sections into clearly labeled subsections (e.g., Design, Sample, Procedure, Analysis). This structure will guide readers through your methodology and findings more effectively and reinforce the manuscript’s technical soundness”.

The recommendations of the review team are accepted. The methods section is merged with the results. This is presented describing separately each of the stages associated with the construction of the instrument and the results obtained in each of them are disclosed. Regarding the use of the alpha and omega coefficients, both results are maintained, since each evaluates different aspects (reliability and internal consistency, respectively), and gives more statical robustness to the results obtained in the exploratory factor analysis.

Regarding the unavailable information, a link to the repository is included, so that the information can be made public, since after reviewing the commitments acquired with the Ethics an Biosafety Committee of the University of Concepción, it was concluded that keeping the information available does not affect the ethical commitments acquired with this entity. The images are auloaded again in two different formats so that it can be visualized

4. Discussion

- **Reviewer 1:** “The discussion should focus on interpreting the results of the inventory rather than emphasizing the broader topic of educational inclusion. It os important to clarify that is a self-perception assessment tool and is not intended for use as an intervention tool”

The discussion section is modified emphasize the results of the pilot study and exploratory factor analysis. The limitations of the study previously identified are maintained and the implications of the use of the instrument in the inclusive educational context are discussed.

Thank you very much for your kind attention. I look forward to hearing from you.

**Dr(c). Carolina Messer Soubelet**

**PhD Program in Mental Health,**

**Facultad de Medicina**

**Universidad de Concepción**

**Email:** [**hmesser@udec.cl**](mailto:hmesser@udec.cl)

**Phone: +56988998521**
