# Supplementary material for: Design, development, and pilot study of a scale for educational professionals to assess theory of mind-related skills regarding children with ASD
Source: PLoS One. 2026 Aug 28;21(8):e0355044. doi: 10.1371/journal.pone.0355044 (PMC13524288; doi:10.1371/journal.pone.0355044)
Supplement: Supplementary file 3 [file pone.0355044.s004.docx]

**January 04, 2026**

**Editor**

**Plos One**

**Of our consideration:**

We are submitting the revised manuscript entitled **“Desingn, Development, and Pilot Study of a Scale for Educational Professionals to Assess Theory of Mind Skills Regarding Children with ASD”,** to consider its publication as a Research Article in Plos One.

The comments made by the review team have been accepted in their entirety. Therefore, in general terms, I would like to inform you that major modifications have been made to the manuscript, which I detail bellow:

- Abstract: Completely revised to ensure consistency with the study presented, emphasizing the psychometric analysis of the instrument.
- Introduction: The wording was adjusted by either deleting unnecessary paragraphs or incorporating relevant information that helps to ensure consistency with the study presented and the research problem addressed.
- Methods: The wording and grammar were reviewed and adjusted.
- Results: Adjusted in exploratory factor analysis by removing the supplementary questions presented in the previous manuscript because they do not contribute to the measurement of ToM skills and serve more as variables that affect the use of ToM. This section also incorporates all the tables associated with the EFA, and it will be up to the editor to indicate which of them will be incorporated into the manuscript at the time of publication.
- Discussion and conclusions: This section completely modified and structured in line with the present study presented, emphasizing the results obtained in the EFA.

Bellow, I provide you with derailed information on the modifications made in accordance with the comments of the review team:

Reviewer 1:

- Comment 1 Abstract: In the abstract it is mentioned: “35 …determining 45 items and three major sections: interpersonal component (which includes the abilities to recognition of emotions, making inferences, and empathic response), intrapersonal component (which includes the ability to identify one’s own thoughts and emotions, reflective ability, planning ability and emotions regulation ability), and a supplementary section containing questions that explore the relationship between work experience with making inference, and the need for training in empathic response.” However, the first EFA yielded 8 factors (line 411), the second analysis was performed with 7 factors (line 432), and this structure was confirmed. The abstract should be revised to reflect the actual factorial structure (7 or 8 factors) found in the analyses. The discrepancy between the theoretical three-section model and the empirical results must be explained. In lines 443–448, it is mentioned that section 2 includes factors 2, 5, 6, and 7 because they belong to intrapersonal dimensions, but a discussion about this is missing. Additionally, the discussion should address why the intrapersonal component expanded from two to four factors. In fact, this comment includes both the results and the discussion, because it was mentioned in the discussion that the third section of the questionnaire could be omitted to improve reliability.

Response: The abstract was modified in its entirety to ensure consistency with the study presented. The EFA was also modified by removing supplementary questions for the reasons stated above, and a five-factor structure is presented.

- Comment 2 Theoretical focus: Lines 63–68 discuss ToM development in children, while the study aims to validate an instrument for adults. Consider removing or shortening this section. Focus the theoretical background on ToM development and assessment in adult populations.

Response: The theoretical focus of the introduction has been adjusted to emphasize the focus of the study presented.

- Comment 3 Conceptual clarification: Lines 79–80: “It includes two subcomponents: 80 cognitive affective ToM and empathic affective ToM.” The phrase may contain a wording issue. Consider “cognitive and affective ToM,” as later you mention “Higher order ToM 86 skills, both cognitive and affective.”

Response: This suggestion is accepted, and the introduction is modified by removing redundant terms.

- Comment 4 Relevance of child instruments: Lines 93–97 refer to ToM instruments for children.
  Since this paper focuses on an adult instrument, these references should be minimized or replaced with examples of adult ToM assessments.

Response: This suggestion is accepted and the introduction is adjusted by removing studies associated with the child population and emphasizing instruments that assess ToM in the adult population, with examples of their use.

- Comment 5 Redundancy: Line 108: It is not necessary to write “Theory of Mind”; “ToM” is sufficient once it has been introduced. Simplify terminology to improve readability.

Response: The grammar of the article has been revised and redundancy has been avoided.

- Comment 6 Literature update: Line 141: “141 used to assess mentalization impairments in psychiatric diagnoses” ToM instruments are now used with healthy adults, not only with individuals with ASD. Include recent references and discuss their relevance for validating ToM in neurotypical populations.

Response: This suggestion is accepted and information on instruments that measure ToM in the adult population is incorporated into the introduction, consistent with the study presented.

- Comment 7 Objectives not achieved: Lines 152–153 and 154–156: The stated purpose and general objective are not fully achieved in the current version. Revise the objectives to align with what was actually accomplished or expand the analysis to fulfill them.

Response: This suggestion is accepted, and wording of the study’s objectives and purpose has been modified.

- Comment 8 Inconsistency in structure: Lines 187–191: The questionnaire was designed with five sections (three interpersonal and two intrapersonal), but results show a different structure, and this is not discussed. Address this discrepancy explicitly in the discussion section.

Response: This suggestion is accepted, and the discussion and conclusions section has been modified to include an analysis of the results obtained in the RFA and their discrepancy with model initially presented.

- Comment 9 Tables and appendices: Table 2 appears unnecessary. Additionally, the EFA factor loadings are missing. Line 413 refers to an appendix that is not provided.
  Remove Table 2 if redundant and include an appendix or supplementary table with the EFA factor loadings. The appendix was mentioned in line 448, but I couldn´t find it.

Response: This suggestion is accepted. Table 2 is removed and an additional paragraph describing this information is added. Regarding the EFA tables, to ensure that reviewers have access to the information, all the tables are included in the manuscript (in addition to being included as an appendix), but it will be up to the editor to decide which of them remain in the manuscript at the time of publication.

- Comment 10 Sample inconsistencies: There were 291 participants, but the analyses use different sample sizes. Explain the reason for varying sample sizes (e.g., missing data, exclusion criteria). If possible, justify whether data imputation was considered.

Response: This suggestion is accepted and an analysis regarding this discrepancy is incorporated. This discrepancy is also incorporated as a limitation of the study, indicating a possible bias in the participants’ responses.

- Comment 11 Factor interpretation: Line 436: Instead of performing a third EFA with the items in factor 1, the work could be to analyze the name of that factor.
  Consider refining the factor’s conceptual interpretation instead of reanalyzing items unnecessarily.

Response: This suggestion is accepted, and information regarding the name of the sections is incorporated, considering a conceptual interpretation of these results.

- Comment 12 Factor composition: How many items are there in each factor?
  Add this information to Table 3 for clarity and completeness.

Response: This suggestion is accepted and a table indicating the number of items contained in each section of the instrument is incorporated.

- Comment 13 Reliability reporting: Line 524: The average reliability (0.63) is reported in the discussion rather than the results. The alpha values for each factor are high, but the overall average is low. Reliability statistics should be reported in the Results section. Clarify how the mean alpha was calculated and explain why the average is lower than individual factor alphas. Also, specify how many items were removed when the complementary section was excluded, and clearly define the final structure (number of sections and items). Finally, the authors suggested that alpha improves if the third section is omitted. Therefore, how many sections and items does the final proposed instrument include?

Response: This suggestion is accepted, and the supplementary questions presented in the previous manuscript are removed from the EFA for the reasons previously stated, thereby ensuring the coherence and consistency of the results presented.

- Overall recommendation: The manuscript requires revisions before it can be considered for publication. The main issues are inconsistencies between the theoretical model and empirical results, incomplete reporting of analyses, and unclear reliability information. Strengthening the discussion and aligning the abstract and results sections with the actual findings would significantly improve the manuscript.

Response: This suggestion is accepted, and a major modifications have been made to the manuscript to address this comment from the reviewer.

Reviewer 3:

- Comment 1 Study objectives: There is some inconsistency in the way the objectives are formulated. In some sections, the main goal is presented as the design, development, and pilot validation of the instrument, while in other passages it is described as assessing the extent to which teachers’ ToM skills influence inclusion in the school community. I recommend unifying and clarifying the central aim of the study, since this affects the overall coherence of the manuscript.

Response: This suggestion is accepted, and modifications are made to the study objectives to ensure consistency with the article presented.

- Comment 2 Expert judgment: The use of five experts with experience in inclusion is valuable; however, for psychometric validation, it would also be advisable to involve specialists in methodology or educational/psychological assessment, who could provide more technical input regarding instrument development and validation. This does not invalidate the process carried out, but it should be acknowledged as a limitation.

Response: This suggestion is accepted and incorporated as a limitation of the study.

- Comment 3 Cognitive interviews: It would be useful to expand the explanation of how these interviews were conducted, what type of questions were asked, and how they contributed to refining the items. This would improve methodological transparency.

Response: This suggestion id accepted, and additional paragraphs explaining how the cognitive interview was conducted have been added to the methods section.

- Comment 4 Writing and clarity: While the manuscript is generally understandable, the English writing contains stylistic issues and at times is difficult to follow, particularly in the introduction. A professional language revision is strongly recommended to ensure clarity and precision.

Response: This suggestion is accepted, and a thorough review of the English writing and wording is being conducted to ensure clarity and precision in the manuscript.

- Comment 5 Discussion: This section could be strengthened by including a deeper reflection on the practical implications of the instrument, its limitations, and next steps.

Response: This suggestion is welcomed and a more detailed analysis of the results obtained and their practical implications is included in the discussion, along with suggestions for future research that could be carried out.

Thank you very much for your kind attention. I look forward to hearing from you.

**Dr(c). Carolina Messer Soubelet**

**PhD Program in Mental Health,**

**Facultad de Medicina**

**Universidad de Concepción**

**Email:** [**hmesser@udec.cl**](mailto:hmesser@udec.cl)

**Phone: +56988998521**
