# Supplementary material for: Design, development, and pilot study of a scale for educational professionals to assess theory of mind-related skills regarding children with ASD
Source: PLoS One. 2026 Aug 28;21(8):e0355044. doi: 10.1371/journal.pone.0355044 (PMC13524288; doi:10.1371/journal.pone.0355044)
Supplement: Supplementary file 4 [file pone.0355044.s005.docx]

**May 18, 2026**

**Editor**

**Plos One**

**Of our consideration:**

We are submitting the fourth revised version of our manuscript, entitled **“Design, Development, and Pilot Study of a Scale for Educational Professionals to Assess Theory of Mind Skills Regarding Children with ASD”,** to consider its publication as a Research Article in Plos One.

We have carefully considered the reviewer’s comments and revised the manuscript accordingly. In this new version, several modifications were made to strengthen the conceptual, methodological, and editorial consistency of this article. These revisions include:

- A full review of the reference list, with the removal of duplicate and uncited references
- A more detailed report of the statistical procedure, including the explicit incorporation of bootstrap procedures alongside the exploratory factor analysis
- The retention of exploratory factor analysis tables in the current version, pending the Editor’s decision regarding which materials should remain in the main text, and which may be more appropriately presented as supplementary material.

Bellow, I provide you with derailed information on the modifications made in accordance with the comments of the reviewer:

- Comment 1 Theoretical Incoherence and Irrelevant Frameworks: The introduction continues to include sections that do not inform the development of the instrument. Specifically, the paragraph regarding neuroimaging (lines 83-88) and the reference to Mentalization-Based Treatment (MBT) (lines 94-98) are extraneous to the educational context of the study. MBT is a clinical psychiatric framework that does not translate to the professional competencies of teachers in a regular classroom. Furthermore, the disproportionate focus on childhood development is unnecessary; the discussion should be redirected toward Theory of Mind (ToM) in adults and neurotypical populations. There is a vast body of current literature and validated instruments for this demographic that has not been adequately integrated.

Response: We appreciate this comment and revised the Introduction accordingly. The paragraph referring to neuroimaging studies and the reference to Mentalization-Based Treatment in school settings were removed to maintain a clearer alignment with the aims and context of the study. In addition, the Introduction was strengthened by incorporating instruments designed to assess Theory of Mind and mentalization in adolescent and adult populations, as well as recent systematic reviews addressing their psychometric properties. This revision also makes clearer that most available instruments were developed for clinical or general research purposes and are not specifically suited to the situated demands of educational practice. At the same time, we maintain that the references to children in the Introduction are limited to the minimum necessary to contextualize the educational relevance of Theory of Mind in relation to students diagnosed with ASD, which is the population that gives specificity to the present instrument.

- Comment 2 Construct Confusion (ToM vs. Inclusive Education): The term "inclusive education" must be removed from the keywords. Possessing ToM skills (a cognitive capacity) does not inherently guarantee inclusive practices or attitudes. The instrument assesses the self-perception of a socio-cognitive skill, not educational inclusion outcomes. Maintaining this conceptual ambiguity invalidates the construct validity of the scale.

Response: We accept the reviewer’s concern and revised the manuscript to reduce potential conceptual ambiguity. Specifically, terms such as “inclusive education” and “inclusive classrooms” were replaced with “schools implementing inclusion-related policies” when referring to the educational context. We agree that the present instrument does not assess inclusion outcomes and that self-reported ToM-related skills should not be interpreted as equivalent to inclusive practices. For this reason, the revised version now frames the instrument more precisely as a measure of perceived socio-cognitive and affective skills related to Theory of Mind in professional educational interactions.

- Comment 3 Methodological and Statistical Deficiencies: Factor Rotation: The use of Varimax rotation has not been adequately justified.

Response: We appreciate this observation and incorporated an explicit justification for the use of varimax rotation in the revised Methods section. In this pilot phase, our aim was to obtain an interpretable simple structure suitable for early-stage scale development. We also added supporting references to prior psychometric studies that used varimax rotation in the initial examination and refinement of newly developed questionnaires. While we acknowledge that other rotational approaches may also be appropriate depending on the analytical purpose, we consider the present choice methodologically defensible for this exploratory stage.

- Comment 4 Methodological and Statistical Deficiencies: Sampling (Line 310): The procedure must be explicitly labeled as convenience sampling. Given the low response rate (10.68%), it does not meet the criteria for simple random sampling.

Response: The manuscript consistently identifies the final sampling strategy as convenience sampling. In the previous version, the text indicated that a simple random selection of institutions was initially attempted; however, because participation depended on institutional authorization and only a limited proportion of institutions agreed to take part, the final sample was ultimately shaped by availability and willingness to participate. For this reason, the revised version makes this point even more explicit in both the Abstract and the Methods section. We agree that the achieved sample does not meet the criteria for simple random sampling, and the manuscript now states this more directly.

- Comment 5 Methodological and Statistical Deficiencies: Factorial Discrepancy: The lack of a thorough discussion regarding the divergence between the initial theoretical model and the multi-factor structure found in the results persists.

Response: We respectfully disagree with this comment, as it does not accurately reflect the content of the previous version of the manuscript. The discrepancy between the initial theoretical model and the multifactorial structure obtained in the exploratory factor analysis had already been explicitly developed in the Discussion and treated as a central interpretive issue. In the previous version, as in the current one, we discussed this divergence in detail, framed it cautiously in view of the pilot nature of the study and the limited sample, and explicitly stated in the Limitations section that replication in broader and more diverse samples is required before drawing stronger conclusions about the factorial structure. Accordingly, we consider that this point had already been fully incorporated in the manuscript prior to the present revision.

- Comment 6 Language Quality and Technical Editing: Despite the authors' claims of a professional review, basic grammatical errors remain that undermine the manuscript's rigor. For instance: Line 228: "an instrument... were developed" (subject-verb agreement error). Line 169: A numbering error in the study stages persists (skipping from stage 3 to 5).

Response: We accept this suggestion. The specific issues identified by the reviewer were corrected in the revised manuscript, including the subject–verb agreement error and the numbering inconsistency in the stages of instrument development. In addition, the manuscript underwent a new revision aimed at improving clarity and formal consistency.

- Comment 7 Keywords: Methodological terms such as "exploratory factor analysis" and "self-report questionnaire" must be removed and replaced with precise thematic descriptors.

Response: We accept this suggestion. The methodological keywords were removed and replaced with thematic descriptors more closely aligned with the substantive focus of the manuscript.

Thank you very much for your kind attention. I look forward to hearing from you.

**Dr(c). Carolina Messer Soubelet**

**PhD Program in Mental Health,**

**Facultad de Medicina**

**Universidad de Concepción**

**Email:** [**hmesser@udec.cl**](mailto:hmesser@udec.cl)

**Phone: +56988998521**
