# Supplementary material for: Design, development, and pilot study of a scale for educational professionals to assess theory of mind-related skills regarding children with ASD
Source: PLoS One. 2026 Aug 28;21(8):e0355044. doi: 10.1371/journal.pone.0355044 (PMC13524288; doi:10.1371/journal.pone.0355044)
Supplement: Supplementary file 5 [file pone.0355044.s006.docx]

**July 04, 2026**

**Editor**

**Plos One**

**Of our consideration:**

We are pleased to submit the revised version of our manuscript, entitled **“Design, Development, and Pilot Study of a Scale for Educational Professionals to Assess Theory of Mind-Related Skills Regarding Children with ASD”,** for consideration as a Research Article in Plos One.

The comments made by the review team have been addressed and the corresponding modifications are detailed below:

**Reviewer 3:** I appreciate the substantial improvements made throughout the review process. The manuscript is now clearer and more consistent in terms of its objectives, theoretical framework, interpretation of findings, and discussion of limitations.

However, some conceptual ambiguity remains regarding the distinction between Theory of Mind itself and self-reported ToM-related skills. I encourage the authors to continue. Emphasizing this distinction throughout the manuscript and to maintain a cautious interpretation of the findings given the exploratory nature of the study.

Response: We appreciated Reviewer 3’s suggestions. We agree that a clear distinction between Theory of Mind as a socio-cognitive construct and the self-reported ToM-related skills assessed by the instrument is essential. In response, we revised the manuscript to emphasize that the instrument does not measure ToM ability directly or through performance-based tasks. Rather, it assesses education professionals’ self-reported ToM-related skills as applied in everyday interactions with students diagnosed with ASD. We also revised the title, objective, discussion, and limitations to maintain a cautious interpretation of the findings, consistent with the exploratory nature of the pilot study.

In the title, the term “Theory of Mind Skills” was replaced with “Theory of Mind-Related Skills” to ensure consistency with the terminology used throughout the manuscript. In the Introduction, a paragraph was added to further clarify the objective of the study:

“However, the instrument was not designed to measure ToM as a latent cognitive ability or performance-based construct. Rather, it assesses professionals’ self-reported ToM-related skills as applied to everyday interactions with students diagnosed with ASD in school settings”.

The wording of the study objective was also revised to indicate that the instrument assesses how education professionals perceive and report the use of their ToM-related skills. In the Discussion, the interpretation of the findings was revised to maintain appropriate caution regarding the preliminary nature of the study, which was based on a limited sample.

In the Result section we have retained all tables associated with the EFA. We defer to the Editor’s judgment regarding which tables should be included in the final published version of the manuscript.

We would also like to clarify that, since the second revision, all data associated with the results reported in the manuscript have been made available through the following link: <https://udeconce-my.sharepoint.com/:f:/g/personal/hmesser_udec_cl/EqazXZ8bzNZMjiQYDQkowBTCguE1AQ05upPDc5eBUbOA?e=XCjzcj>. We would be grateful if the Editor could confirm whether the link provides appropriate access to the data or whether a new link should be generated.

Finally, we have added acknowledgment of the funding that supports Dr. Riffo's work. This addition does not modify the content, results, or interpretation of the manuscript.

Thank you very much for your kind attention. We look forward to hearing from you.

**Dr(c). Carolina Messer Soubelet**

**PhD Program in Mental Health,**

**Facultad de Medicina**

**Universidad de Concepción**

**Email:** [**hmesser@udec.cl**](mailto:hmesser@udec.cl)

**Phone: +56988998521**
