# Supplementary material for: Design, development, and pilot study of a scale for educational professionals to assess theory of mind-related skills regarding children with ASD
Source: PLoS One. 2026 Aug 28;21(8):e0355044. doi: 10.1371/journal.pone.0355044 (PMC13524288; doi:10.1371/journal.pone.0355044)
Supplement: Peer review history

# Peer review: Decision Letter 0

**DOI:** 10.1371/journal.pone.0355044.r001

**Authors:** Leire Aperribai

31 May 2025

Dear Dr. Messer Soubelet,

Thank you for submitting your manuscript to PLOS ONE. After careful consideration, we feel that it has merit but does not fully meet PLOS ONE’s publication criteria as it currently stands. Therefore, we invite you to submit a revised version of the manuscript that addresses the points raised during the review process.

Please submit your revised manuscript by Jul 15 2025 11:59PM. If you will need more time than this to complete your revisions, please reply to this message or contact the journal office at plosone@plos.org. When you're ready to submit your revision, log on to https://www.editorialmanager.com/pone/ and select the 'Submissions Needing Revision' folder to locate your manuscript file.

- A rebuttal letter that responds to each point raised by the academic editor and reviewer(s). You should upload this letter as a separate file labeled 'Response to Reviewers'.
- A marked-up copy of your manuscript that highlights changes made to the original version. You should upload this as a separate file labeled 'Revised Manuscript with Track Changes'.
- An unmarked version of your revised paper without tracked changes. You should upload this as a separate file labeled 'Manuscript'.

If you would like to make changes to your financial disclosure, please include your updated statement in your cover letter. Guidelines for resubmitting your figure files are available below the reviewer comments at the end of this letter.

If applicable, we recommend that you deposit your laboratory protocols in protocols.io to enhance the reproducibility of your results. Protocols.io assigns your protocol its own identifier (DOI) so that it can be cited independently in the future. For instructions see: https://journals.plos.org/plosone/s/submission-guidelines#loc-laboratory-protocols. Additionally, PLOS ONE offers an option for publishing peer-reviewed Lab Protocol articles, which describe protocols hosted on protocols.io. Read more information on sharing protocols at https://plos.org/protocols?utm_medium=editorial-email&utm_source=authorletters&utm_campaign=protocols.

We look forward to receiving your revised manuscript.

Kind regards,

Leire Aperribai

Academic Editor

PLOS ONE

Journal Requirements:

When submitting your revision, we need you to address these additional requirements.

1. Please ensure that your manuscript meets PLOS ONE's style requirements, including those for file naming. The PLOS ONE style templates can be found at https://journals.plos.org/plosone/s/file?id=wjVg/PLOSOne_formatting_sample_main_body.pdf and https://journals.plos.org/plosone/s/file?id=ba62/PLOSOne_formatting_sample_title_authors_affiliations.pdf

2. Please include your full ethics statement in the ‘Methods’ section of your manuscript file. In your statement, please include the full name of the IRB or ethics committee who approved or waived your study, as well as whether or not you obtained informed written or verbal consent. If consent was waived for your study, please include this information in your statement as well.

3. We note that you have indicated that there are restrictions to data sharing for this study. For studies involving human research participant data or other sensitive data, we encourage authors to share de-identified or anonymized data. However, when data cannot be publicly shared for ethical reasons, we allow authors to make their data sets available upon request. For information on unacceptable data access restrictions, please see http://journals.plos.org/plosone/s/data-availability#loc-unacceptable-data-access-restrictions.

Before we proceed with your manuscript, please address the following prompts:

a) If there are ethical or legal restrictions on sharing a de-identified data set, please explain them in detail (e.g., data contain potentially identifying or sensitive patient information, data are owned by a third-party organization, etc.) and who has imposed them (e.g., a Research Ethics Committee or Institutional Review Board, etc.). Please also provide contact information for a data access committee, ethics committee, or other institutional body to which data requests may be sent.

b) If there are no restrictions, please upload the minimal anonymized data set necessary to replicate your study findings to a stable, public repository and provide us with the relevant URLs, DOIs, or accession numbers. Please see http://www.bmj.com/content/340/bmj.c181.long for guidelines on how to de-identify and prepare clinical data for publication. For a list of recommended repositories, please see https://journals.plos.org/plosone/s/recommended-repositories. You also have the option of uploading the data as Supporting Information files, but we would recommend depositing data directly to a data repository if possible.

Please update your Data Availability statement in the submission form accordingly.

4. When completing the data availability statement of the submission form, you indicated that you will make your data available on acceptance. We strongly recommend all authors decide on a data sharing plan before acceptance, as the process can be lengthy and hold up publication timelines. Please note that, though access restrictions are acceptable now, your entire data will need to be made freely accessible if your manuscript is accepted for publication. This policy applies to all data except where public deposition would breach compliance with the protocol approved by your research ethics board. If you are unable to adhere to our open data policy, please kindly revise your statement to explain your reasoning and we will seek the editor's input on an exemption. Please be assured that, once you have provided your new statement, the assessment of your exemption will not hold up the peer review process.

5. Please amend your authorship list in your manuscript file to include author Carolina Messer Soubelet.

6. Please amend the manuscript submission data (via Edit Submission) to include author Carolina Messer.

7. We are unable to open your Figure [Copia de etapas instrument.eps, Copia de ToM dimension.eps]. Please kindly revise as necessary and re-upload.

8. Please remove all personal information, ensure that the data shared are in accordance with participant consent, and re-upload a fully anonymized data set.

Note: spreadsheet columns with personal information must be removed and not hidden as all hidden columns will appear in the published file.

Additional guidance on preparing raw data for publication can be found in our Data Policy (https://journals.plos.org/plosone/s/data-availability#loc-human-research-participant-data-and-other-sensitive-data) and in the following article: http://www.bmj.com/content/340/bmj.c181.long.

9. Please include captions for your Supporting Information files at the end of your manuscript, and update any in-text citations to match accordingly. Please see our Supporting Information guidelines for more information: http://journals.plos.org/plosone/s/supporting-information.

Additional Editor Comments :

Dear author,

After considering reviewers decisions, I would like to tell you that we consider the manuscript for a major revision.

Therefore, we strongly recommend to read carefully reviewers suggestions and follow all of them to a second submission.

Thank you for considering PLOS One for your submission.

Kind regards

Leire Aperribai PhD

Academic Editor

[Note: HTML markup is below. Please do not edit.]

Reviewers' comments:

Reviewer's Responses to Questions

**Comments to the Author**

1. Is the manuscript technically sound, and do the data support the conclusions?

Reviewer #1: Partly

Reviewer #2: Yes

**********

2. Has the statistical analysis been performed appropriately and rigorously?

Reviewer #1: No

Reviewer #2: Yes

**********

3. Have the authors made all data underlying the findings in their manuscript fully available?

The PLOS Data policy

Reviewer #1: No

Reviewer #2: No

**********

4. Is the manuscript presented in an intelligible fashion and written in standard English?

Reviewer #1: No

Reviewer #2: Yes

**********

Reviewer #1: Abstract

The abstract begins by discussing children with ASD, but the main focus of the manuscript is an instrument, not a study involving children.

Lines 32–33: There is a lack of alignment between the title (which refers to professionals) and the objective stated in the abstract (which refers to children).

Lines 34–35: A second objective is introduced: “to present the design, construction, and validation of an instrument that assesses the use of Theory of Mind skills in educational settings, along with the results from its first pilot study.” This is unclear. Is the instrument intended for use with children or with teachers in educational settings? Furthermore, why do the authors refer to “validation” if this is only a pilot study?

The number of items included in the instrument is not mentioned. Also, a correlation of .37 is relatively weak and should be interpreted cautiously. Additionally, it is unclear why correlations between items are reported.

The results of the exploratory factor analysis are not mentioned in the abstract, despite being crucial for establishing the internal structure of the instrument.

Introduction

The first pages of the introduction address a wide range of Theory of Mind (ToM) topics: definitions, development, neurological correlates, classifications, ASD, and ToM deficits in ASD. However, these topics are not directly related to the stated objective of the manuscript.

The introduction should be rewritten to provide a focused rationale for the construction of the instrument.

Additionally, there is a shift in focus: the manuscript discusses children with ASD and educational communities, but the emphasis should be on teachers, as suggested by the title.

Method

The Method section should follow a clear structure with subsections such as Participants, Instrument, Procedure, and Data Analysis. Currently, the narrative style makes the content difficult to follow.

The exploratory factor analysis should include all items, not just dimensions.

The authors should choose one reliability index—either Cronbach’s alpha or McDonald’s omega—and justify their choice.

The raw data are not available in a public repository. Do the authors have any concerns or limitations regarding data sharing?

Results

The presentation of the results should be improved by clearly distinguishing the different stages of the instrument’s development. This could be achieved through subheadings and brief descriptions of each phase.

Greater emphasis should be placed on the application of the instrument to the sample of 291 participants.

The figures are not viewable; only a dark square appears. This must be corrected to allow proper evaluation of the data.

Discussion

The discussion should focus on interpreting the results of the inventory rather than emphasizing the broader topic of educational inclusion.

It is important to clarify that this is a self-perception assessment tool and is not intended for use as an intervention tool.

Reviewer #2: The manuscript addresses a highly relevant topic, and the overall approach is thoughtful and engaging. However, several areas require attention to enhance clarity, coherence, and precision.

First, the Abstract would benefit from greater terminological specificity. The phrase “use of theory of mind skills” is ambiguous: it is unclear whether the instrument assesses children’s ability to apply Theory of Mind (ToM) in educational contexts or teachers’ perceptions of those skills. Similarly, the statement that the instrument “assesses the use of theory of mind skills in educational settings” does not specify whether the subjects of assessment are teachers or children with ToM-related difficulties. I recommend explicitly identifying the target population. Finally, the Abstract inconsistently mixes present and past tenses (e.g., “presents” vs. “was administered”); a uniform past-tense narrative would align better with standard scientific conventions.

In the Introduction, I suggest a comprehensive language review to improve readability. In particular, the sentence beginning “An implicit one…” (line 76) is structurally convoluted and suffers from unclear subject-verb agreement; splitting it into two sentences and clearly defining each clause’s subject would significantly aid comprehension. Additionally, several passages (e.g., line 136) are overly long and could be divided into shorter, more focused sentences. There is also a formatting issue between lines 211 and 212, where an opening quotation mark is not closed, and a period is missing at the end of the sentence on line 239; please correct these errors.

To improve the manuscript’s organization, I recommend dividing the Methods and Results sections into clearly labeled subsections (e.g., Design, Sample, Procedure, Analysis). This structure will guide readers through your methodology and findings more effectively and reinforce the manuscript’s technical soundness. Overall, addressing these points will strengthen the presentation and ensure that the data fully support your conclusions.

**********

what does this mean?). If published, this will include your full peer review and any attached files.

If you choose “no”, your identity will remain anonymous but your review may still be made public.

**Do you want your identity to be public for this peer review?**  For information about this choice, including consent withdrawal, please see our Privacy Policy

Reviewer #1: No

Reviewer #2: No

**********

[NOTE: If reviewer comments were submitted as an attachment file, they will be attached to this email and accessible via the submission site. Please log into your account, locate the manuscript record, and check for the action link "View Attachments". If this link does not appear, there are no attachment files.]

While revising your submission, please upload your figure files to the Preflight Analysis and Conversion Engine (PACE) digital diagnostic tool, https://pacev2.apexcovantage.com/. PACE helps ensure that figures meet PLOS requirements. To use PACE, you must first register as a user. Registration is free. Then, login and navigate to the UPLOAD tab, where you will find detailed instructions on how to use the tool. If you encounter any issues or have any questions when using PACE, please email PLOS at figures@plos.org. Please note that Supporting Information files do not need this step.

# Author comment: Author response to Decision Letter 1

**DOI:** 10.1371/journal.pone.0355044.r002

14 Jul 2025

the reviewers' comments are accepted and responded to in a letter to the editor. The manuscript has been modified according to the reviewers' indications. The abstract and introduction have been modified to focus on the construction of the instrument and the methods section has been arranged to give it a more understandable structure.

Attachment Submitted filename: rebuttal letter .docx

# Peer review: Decision Letter 1

**DOI:** 10.1371/journal.pone.0355044.r003

**Authors:** Leire Aperribai

7 Aug 2025

Dear Dr. Messer-Soubelet,

Thank you for submitting your manuscript to PLOS ONE. After careful consideration, we feel that it has merit but does not fully meet PLOS ONE’s publication criteria as it currently stands. Therefore, we invite you to submit a revised version of the manuscript that addresses the points raised during the review process.

Please submit your revised manuscript by Sep 21 2025 11:59PM. If you will need more time than this to complete your revisions, please reply to this message or contact the journal office at plosone@plos.org. When you're ready to submit your revision, log on to https://www.editorialmanager.com/pone/ and select the 'Submissions Needing Revision' folder to locate your manuscript file.

- A rebuttal letter that responds to each point raised by the academic editor and reviewer(s). You should upload this letter as a separate file labeled 'Response to Reviewers'.
- A marked-up copy of your manuscript that highlights changes made to the original version. You should upload this as a separate file labeled 'Revised Manuscript with Track Changes'.
- An unmarked version of your revised paper without tracked changes. You should upload this as a separate file labeled 'Manuscript'.

If you would like to make changes to your financial disclosure, please include your updated statement in your cover letter. Guidelines for resubmitting your figure files are available below the reviewer comments at the end of this letter.

If applicable, we recommend that you deposit your laboratory protocols in protocols.io to enhance the reproducibility of your results. Protocols.io assigns your protocol its own identifier (DOI) so that it can be cited independently in the future. For instructions see: https://journals.plos.org/plosone/s/submission-guidelines#loc-laboratory-protocols. Additionally, PLOS ONE offers an option for publishing peer-reviewed Lab Protocol articles, which describe protocols hosted on protocols.io. Read more information on sharing protocols at https://plos.org/protocols?utm_medium=editorial-email&utm_source=authorletters&utm_campaign=protocols.

We look forward to receiving your revised manuscript.

Kind regards,

Leire Aperribai

Academic Editor

PLOS ONE

Journal Requirements:

If the reviewer comments include a recommendation to cite specific previously published works, please review and evaluate these publications to determine whether they are relevant and should be cited. There is no requirement to cite these works unless the editor has indicated otherwise.

Additional Editor Comment:

Thank you for the submission made.

As reviewer 1 has mentioned there are still some issues that should improve. Therefore, I strongly suggest authors to follow one by one all the recommendations made by reviewer 1.

I wish authors can fulfil all the requirements and submit again the manuscript, since I consider that the study is of importance in the field.

Thank you in advance.

[Note: HTML markup is below. Please do not edit.]

Reviewers' comments:

Reviewer's Responses to Questions

**Comments to the Author**

Reviewer #1: (No Response)

Reviewer #2: All comments have been addressed

**********

2. Is the manuscript technically sound, and do the data support the conclusions?

Reviewer #1: Yes

Reviewer #2: (No Response)

**********

3. Has the statistical analysis been performed appropriately and rigorously?

Reviewer #1: No

Reviewer #2: (No Response)

**********

4. Have the authors made all data underlying the findings in their manuscript fully available?

The PLOS Data policy

Reviewer #1: Yes

Reviewer #2: (No Response)

**********

5. Is the manuscript presented in an intelligible fashion and written in standard English?

Reviewer #1: Yes

Reviewer #2: (No Response)

**********

Reviewer #1: Title

I suggest make a few changes in title to make clear that is a scale assessing theory of mind for professionals specifically about children with ASD, for example: Design, Development, and Pilot Study of a Scale for Educational Professionals to Assess Theory of Mind Skills Regarding Children with ASD. Short Title also must be specified.

Abstract

It is recommended to include the overall KMO value of the Exploratory Factor Analysis (EFA) for all items in the abstract to strengthen the methodological rigor.

Introduction

The previous suggestions have been addressed, and the direction of the study is now clearer.

However, some aspects could still be improved.

Line 105:

Original: “Taking into account the above, the need arises to construct an instrument that measures how teachers and professionals linked to Education use their ToM skills in children diagnosed with ASD.”

Suggested revision:

“Given the above, there is a need to develop an instrument that measures how education professionals apply their Theory of Mind (ToM) skills when working with children diagnosed with ASD.”

Line 107:

“The aim is to study the incidence of ToM on the school inclusion of children diagnosed with ASD.” This line could be eliminated as it is redundant or insufficiently developed in the current version.

Line 134:

“Considering the principles of ToM, the idea of studying between the use of ToM skills by education professionals and their effect on the school inclusion of children diagnosed with ASD was proposed.” This paragraph could be eliminated since the idea is not further elaborated in the new version of the manuscript.

Method and Results

Line 67: It is not standard to combine "Methods and Results" into a single section. It is recommended to separate them. For reference, consult: Goel, S., Angeli, F., Singla, N., & Ruwaard, D. (2016). Development and validation of the motivations for selection of medical study (MSMS) questionnaire in India. PLOS ONE, 11(12), e0164581.

Tables and Figures:

There are too many tables and figures. Table 1 may be removed, and its content integrated into the main text for conciseness.

Table 2: What does “Inter.” mean? Please define all abbreviations.

Table 3: The first row needs correction, What does “260 wor exp” mean? What is “244 years of perm.”? Please clarify these abbreviations or rephrase the variables for clarity.

Line 402:

“An exploratory factor analysis was conducted using maximum likelihood extraction and varimax.” If the theoretical model proposes five dimensions, and the intention is to test them, a Confirmatory Factor Analysis (CFA) is more appropriate. If EFA is retained, it should be explained why five separate EFAs were conducted instead of one comprehensive EFA with the 45 items. While conducting EFA by dimension is sometimes acceptable, results must be interpreted cautiously since inter-dimensional interactions may influence factor structure.

Line 403:

“Prior to that, for each evaluated TM dimension…” Confirm whether “TM” refers to “ToM”. If so, please correct it for consistency.

KMO Reporting: If five EFAs were conducted (one per dimension), then five KMO values would result. However, the global KMO for the entire scale (all 45 items) must also be reported.

Line 464:

The authors report both alpha and omega coefficients. Both are reliability indices, but the choice depends on the data characteristics. It is recommended to report the most appropriate reliability index based on your data. For consult: Peters, G. J. Y. (2014). The alpha and the omega of scale reliability and validity: Why and how to abandon Cronbach's alpha and the route towards more comprehensive assessment of scale quality. European Health Psychologist, 16(2), 56–69.

Tables: Correct the name of the analysis in table titles: use Exploratory Factor Analysis (EFA). Table 4: Correct the p-values and review the degrees of freedom (df). Why are they so variable? Tables 6, 7, and 8: Include the labels Factor 1 and Factor 2 in the column headings.

Limitations

It is recommended to mention explicitly that this is a pilot study, which explains why the sample does not include the usual minimum of five participants per item.

If the EFAs by dimension are retained, include a cautionary note about interpreting results from separate EFAs instead of a unified analysis.

Reviewer #2: (No Response)

**********

what does this mean?). If published, this will include your full peer review and any attached files.

If you choose “no”, your identity will remain anonymous but your review may still be made public.

**Do you want your identity to be public for this peer review?**  For information about this choice, including consent withdrawal, please see our Privacy Policy

Reviewer #1: **Yes:** Bernarda Téllez Alanís

Reviewer #2: No

**********

[NOTE: If reviewer comments were submitted as an attachment file, they will be attached to this email and accessible via the submission site. Please log into your account, locate the manuscript record, and check for the action link "View Attachments". If this link does not appear, there are no attachment files.]

While revising your submission, please upload your figure files to the Preflight Analysis and Conversion Engine (PACE) digital diagnostic tool, https://pacev2.apexcovantage.com/. PACE helps ensure that figures meet PLOS requirements. To use PACE, you must first register as a user. Registration is free. Then, login and navigate to the UPLOAD tab, where you will find detailed instructions on how to use the tool. If you encounter any issues or have any questions when using PACE, please email PLOS at figures@plos.org. Please note that Supporting Information files do not need this step.

# Author comment: Author response to Decision Letter 2

**DOI:** 10.1371/journal.pone.0355044.r004

22 Sep 2025

All suggestions made by the reviewer have been accepted and included in the manuscript.

# Peer review: Decision Letter 2

**DOI:** 10.1371/journal.pone.0355044.r005

**Authors:** Leire Aperribai

27 Oct 2025

Dear Dr. Messer-Soubelet,

Thank you for submitting your manuscript to PLOS ONE. After careful consideration, we feel that it has merit but does not fully meet PLOS ONE’s publication criteria as it currently stands. Therefore, we invite you to submit a revised version of the manuscript that addresses the points raised during the review process.

Please submit your revised manuscript by Dec 11 2025 11:59PM. If you will need more time than this to complete your revisions, please reply to this message or contact the journal office at plosone@plos.org. When you're ready to submit your revision, log on to https://www.editorialmanager.com/pone/ and select the 'Submissions Needing Revision' folder to locate your manuscript file.

- A rebuttal letter that responds to each point raised by the academic editor and reviewer(s). You should upload this letter as a separate file labeled 'Response to Reviewers'.
- A marked-up copy of your manuscript that highlights changes made to the original version. You should upload this as a separate file labeled 'Revised Manuscript with Track Changes'.
- An unmarked version of your revised paper without tracked changes. You should upload this as a separate file labeled 'Manuscript'.

If you would like to make changes to your financial disclosure, please include your updated statement in your cover letter. Guidelines for resubmitting your figure files are available below the reviewer comments at the end of this letter.

If applicable, we recommend that you deposit your laboratory protocols in protocols.io to enhance the reproducibility of your results. Protocols.io assigns your protocol its own identifier (DOI) so that it can be cited independently in the future. For instructions see: https://journals.plos.org/plosone/s/submission-guidelines#loc-laboratory-protocols. Additionally, PLOS ONE offers an option for publishing peer-reviewed Lab Protocol articles, which describe protocols hosted on protocols.io. Read more information on sharing protocols at https://plos.org/protocols?utm_medium=editorial-email&utm_source=authorletters&utm_campaign=protocols.

We look forward to receiving your revised manuscript.

Kind regards,

Leire Aperribai

Academic Editor

PLOS ONE

Journal Requirements:

If the reviewer comments include a recommendation to cite specific previously published works, please review and evaluate these publications to determine whether they are relevant and should be cited. There is no requirement to cite these works unless the editor has indicated otherwise.

Additional Editor Comments :

Dear Carolina Messer-Soubelet,

After reviewing the latest version of the manuscript, Reviewer 1 has recommended a Major Revision and Reviewer 2 a Minor Revision.

Please, take into consideration all the recommendations made by both reviewers, as we understand that they did valuable contributions to the manuscript. We encourage you to apply these recommendations and to resend the manuscript to the journal.

Thank you for attention.

Kind regards,

Leire Aperribai PhD

[Note: HTML markup is below. Please do not edit.]

Reviewers' comments:

Reviewer's Responses to Questions

**Comments to the Author**

Reviewer #1: (No Response)

Reviewer #3: (No Response)

**********

2. Is the manuscript technically sound, and do the data support the conclusions?

Reviewer #1: Partly

Reviewer #3: Partly

**********

3. Has the statistical analysis been performed appropriately and rigorously?

Reviewer #1: No

Reviewer #3: Yes

**********

4. Have the authors made all data underlying the findings in their manuscript fully available?

The PLOS Data policy

Reviewer #1: Yes

Reviewer #3: Yes

**********

5. Is the manuscript presented in an intelligible fashion and written in standard English?

Reviewer #1: No

Reviewer #3: No

**********

Reviewer #1: The authors made corrections, but not in a complete way. Several methodological and conceptual inconsistencies remain. Below are specific observations and recommendations for improvement.

Comment 1 – Abstract

In the abstract it is mentioned:

“35 …determining 45 items and three major sections: interpersonal component (which

36 includes the abilities to recognition of emotions, making inferences, and empathic response),

37 intrapersonal component (which includes the ability to identify one’s own thoughts and

38 emotions, reflective ability, planning ability and emotions regulation ability), and a

39 supplementary section containing questions that explore the relationship between work

40 experience with making inference, and the need for training in empathic response.”

However, the first EFA yielded 8 factors (line 411), the second analysis was performed with 7 factors (line 432), and this structure was confirmed.

The abstract should be revised to reflect the actual factorial structure (7 or 8 factors) found in the analyses. The discrepancy between the theoretical three-section model and the empirical results must be explained. In lines 443–448, it is mentioned that section 2 includes factors 2, 5, 6, and 7 because they belong to intrapersonal dimensions, but a discussion about this is missing. Additionally, the discussion should address why the intrapersonal component expanded from two to four factors. In fact, this comment includes both the results and the discussion, because it was mentioned in the discussion that the third section of the questionnaire could be omitted to improve reliability.

Comment 2 – Theoretical focus

Lines 63–68 discuss ToM development in children, while the study aims to validate an instrument for adults.

Consider removing or shortening this section. Focus the theoretical background on ToM development and assessment in adult populations.

Comment 3 – Conceptual clarification

Lines 79–80: “It includes two subcomponents: 80 cognitive affective ToM and empathic affective ToM.”

The phrase may contain a wording issue. Consider “cognitive and affective ToM,” as later you mention “Higher order ToM 86 skills, both cognitive and affective.”

Comment 4 – Relevance of child instruments

Lines 93–97 refer to ToM instruments for children.

Since this paper focuses on an adult instrument, these references should be minimized or replaced with examples of adult ToM assessments.

Comment 5 – Redundancy

Line 108: It is not necessary to write “Theory of Mind”; “ToM” is sufficient once it has been introduced.

Simplify terminology to improve readability.

Comment 6 – Literature update

Line 141: “141 used to assess mentalization impairments in psychiatric diagnoses”

ToM instruments are now used with healthy adults, not only with individuals with ASD.

Include recent references and discuss their relevance for validating ToM in neurotypical populations.

Comment 7 – Objectives not achieved

Lines 152–153 and 154–156: The stated purpose and general objective are not fully achieved in the current version.

Revise the objectives to align with what was actually accomplished or expand the analysis to fulfill them.

Comment 8 – Inconsistency in structure

Lines 187–191: The questionnaire was designed with five sections (three interpersonal and two intrapersonal), but results show a different structure, and this is not discussed.

Address this discrepancy explicitly in the discussion section.

Comment 9 – Tables and appendices

Table 2 appears unnecessary. Additionally, the EFA factor loadings are missing. Line 413 refers to an appendix that is not provided.

Remove Table 2 if redundant and include an appendix or supplementary table with the EFA factor loadings. The appendix was mentioned in line 448, but I couldn´t find it.

Comment 10 – Sample inconsistencies

There were 291 participants, but the analyses use different sample sizes.

Explain the reason for varying sample sizes (e.g., missing data, exclusion criteria). If possible, justify whether data imputation was considered.

Comment 11 – Factor interpretation

Line 436: Instead of performing a third EFA with the items in factor 1, the work could be to analyze the name of that factor.

Consider refining the factor’s conceptual interpretation instead of reanalyzing items unnecessarily.

Comment 12 – Factor composition

How many items are there in each factor?

Add this information to Table 3 for clarity and completeness.

Comment 13 – Reliability reporting

Line 524: The average reliability (0.63) is reported in the discussion rather than the results. The alpha values for each factor are high, but the overall average is low.

Reliability statistics should be reported in the Results section. Clarify how the mean alpha was calculated and explain why the average is lower than individual factor alphas. Also, specify how many items were removed when the complementary section was excluded, and clearly define the final structure (number of sections and items). Finally, the authors suggested that alpha improves if the third section is omitted. Therefore, how many sections and items does the final proposed instrument include?

Overall recommendation:

The manuscript requires revisions before it can be considered for publication. The main issues are inconsistencies between the theoretical model and empirical results, incomplete reporting of analyses, and unclear reliability information. Strengthening the discussion and aligning the abstract and results sections with the actual findings would significantly improve the manuscript.

Reviewer #3: I would like to thank the authors for the opportunity to review this manuscript and to commend them for addressing such an important and underexplored topic. The development of an instrument aimed at evaluating Theory of Mind (ToM) skills in education professionals working with students with ASD is highly relevant, both academically and in applied educational contexts, particularly within the framework of inclusive education policies in Chile and the region.

Main strengths

- The study focuses on a novel and relevant area, linking Theory of Mind, inclusive education, and teaching practice.

- The methodology follows appropriate steps: expert judgment, cognitive interviews, pilot testing with a large sample, and exploratory factor analysis using robust statistical criteria (KMO, Bartlett’s test, Horn’s parallel analysis).

- The instrument shows clear potential to become a useful tool in the educational field.

Aspects requiring clarification or revision

1. Study objectives: There is some inconsistency in the way the objectives are formulated. In some sections, the main goal is presented as the design, development, and pilot validation of the instrument, while in other passages it is described as assessing the extent to which teachers’ ToM skills influence inclusion in the school community. I recommend unifying and clarifying the central aim of the study, since this affects the overall coherence of the manuscript.

2. Expert judgment: The use of five experts with experience in inclusion is valuable; however, for psychometric validation, it would also be advisable to involve specialists in methodology or educational/psychological assessment, who could provide more technical input regarding instrument development and validation. This does not invalidate the process carried out, but it should be acknowledged as a limitation.

3. Cognitive interviews: It would be useful to expand the explanation of how these interviews were conducted, what type of questions were asked, and how they contributed to refining the items. This would improve methodological transparency.

4. Writing and clarity: While the manuscript is generally understandable, the English writing contains stylistic issues and at times is difficult to follow, particularly in the introduction. A professional language revision is strongly recommended to ensure clarity and precision.

5. Discussion: This section could be strengthened by including a deeper reflection on the practical implications of the instrument, its limitations, and next steps.

Conclusion

Overall, this manuscript represents a valuable and original contribution. The methodological work is largely sound, though with some limitations that should be recognized. I suggest acceptance after minor to moderate revisions, focusing on clarifying objectives, providing further methodological detail, and improving the writing quality.

**********

what does this mean?). If published, this will include your full peer review and any attached files.

If you choose “no”, your identity will remain anonymous but your review may still be made public.

**Do you want your identity to be public for this peer review?**  For information about this choice, including consent withdrawal, please see our Privacy Policy

Reviewer #1: No

Reviewer #3: No

**********

[NOTE: If reviewer comments were submitted as an attachment file, they will be attached to this email and accessible via the submission site. Please log into your account, locate the manuscript record, and check for the action link "View Attachments". If this link does not appear, there are no attachment files.]

To ensure your figures meet our technical requirements, please review our figure guidelines: https://journals.plos.org/plosone/s/figures

You may also use PLOS’s free figure tool, NAAS, to help you prepare publication quality figures: https://journals.plos.org/plosone/s/figures#loc-tools-for-figure-preparation.

NAAS will assess whether your figures meet our technical requirements by comparing each figure against our figure specifications.

# Author comment: Author response to Decision Letter 3

**DOI:** 10.1371/journal.pone.0355044.r006

6 Jan 2026

the reviewers' comments are accepted and responded to in a letter to the editor.

Attachment Submitted filename: Response to Reviewers.docx

# Peer review: Decision Letter 3

**DOI:** 10.1371/journal.pone.0355044.r007

**Authors:** Leire Aperribai

9 Feb 2026

Dear Dr. Messer-Soubelet,

plosone@plos.org. When you're ready to submit your revision, log on to https://www.editorialmanager.com/pone/ and select the 'Submissions Needing Revision' folder to locate your manuscript file.

- A letter that responds to each point raised by the academic editor and reviewer(s). You should upload this letter as a separate file labeled 'Response to Reviewers'.
- A marked-up copy of your manuscript that highlights changes made to the original version. You should upload this as a separate file labeled 'Revised Manuscript with Track Changes'.
- An unmarked version of your revised paper without tracked changes. You should upload this as a separate file labeled 'Manuscript'.

If you would like to make changes to your financial disclosure, please include your updated statement in your cover letter. Guidelines for resubmitting your figure files are available below the reviewer comments at the end of this letter.

If applicable, we recommend that you deposit your laboratory protocols in protocols.io to enhance the reproducibility of your results. Protocols.io assigns your protocol its own identifier (DOI) so that it can be cited independently in the future. For instructions see: https://journals.plos.org/plosone/s/submission-guidelines#loc-laboratory-protocols. Additionally, PLOS ONE offers an option for publishing peer-reviewed Lab Protocol articles, which describe protocols hosted on protocols.io. Read more information on sharing protocols at https://plos.org/protocols?utm_medium=editorial-email&utm_source=authorletters&utm_campaign=protocols.

We look forward to receiving your revised manuscript.

Kind regards,

Leire Aperribai PhD

Academic Editor

PLOS One

Journal Requirements:

If the reviewer comments include a recommendation to cite specific previously published works, please review and evaluate these publications to determine whether they are relevant and should be cited. There is no requirement to cite these works unless the editor has indicated otherwise.

Please review your reference list to ensure that it is complete and correct. If you have cited papers that have been retracted, please include the rationale for doing so in the manuscript text, or remove these references and replace them with relevant current references. Any changes to the reference list should be mentioned in the rebuttal letter that accompanies your revised manuscript. If you need to cite a retracted article, indicate the article’s retracted status in the References list and also include a citation and full reference for the retraction notice.

[Note: HTML markup is below. Please do not edit.]

Reviewers' comments:

Reviewer's Responses to Questions

**Comments to the Author**

Reviewer #3: (No Response)

**********

2. Is the manuscript technically sound, and do the data support the conclusions?

Reviewer #3: Partly

**********

3. Has the statistical analysis been performed appropriately and rigorously?

Reviewer #3: No

**********

4. Have the authors made all data underlying the findings in their manuscript fully available?

The PLOS Data policy

Reviewer #3: Yes

**********

5. Is the manuscript presented in an intelligible fashion and written in standard English?

Reviewer #3: No

**********

Reviewer #3: General Summary: Upon reviewing the revised manuscript, it is evident that several critical conceptual and methodological inconsistencies previously identified remain unresolved. While the authors have implemented minor editorial changes, the internal logic of the study remains structurally weak. The manuscript fails to establish a rigorous link between the theoretical framework, the professional context of the subjects, and the psychometric operationalization of the instrument.

Specific Comments:

- Theoretical Incoherence and Irrelevant Frameworks: The introduction continues to include sections that do not inform the development of the instrument. Specifically, the paragraph regarding neuroimaging (lines 83-88) and the reference to Mentalization-Based Treatment (MBT) (lines 94-98) are extraneous to the educational context of the study. MBT is a clinical psychiatric framework that does not translate to the professional competencies of teachers in a regular classroom. Furthermore, the disproportionate focus on childhood development is unnecessary; the discussion should be redirected toward Theory of Mind (ToM) in adults and neurotypical populations. There is a vast body of current literature and validated instruments for this demographic that has not been adequately integrated.

- Construct Confusion (ToM vs. Inclusive Education): The term "inclusive education" must be removed from the keywords. Possessing ToM skills (a cognitive capacity) does not inherently guarantee inclusive practices or attitudes. The instrument assesses the self-perception of a socio-cognitive skill, not educational inclusion outcomes. Maintaining this conceptual ambiguity invalidates the construct validity of the scale.

- Methodological and Statistical Deficiencies:

-- Factor Rotation: The use of Varimax rotation has not been adequately justified.

-- Sampling (Line 310): The procedure must be explicitly labeled as convenience sampling. Given the low response rate (10.68%), it does not meet the criteria for simple random sampling.

-- Factorial Discrepancy: The lack of a thorough discussion regarding the divergence between the initial theoretical model and the multi-factor structure found in the results persists.

- Language Quality and Technical Editing: Despite the authors' claims of a professional review, basic grammatical errors remain that undermine the manuscript's rigor. For instance:

Line 228: "an instrument... were developed" (subject-verb agreement error).

Line 169: A numbering error in the study stages persists (skipping from stage 3 to 5).

- Keywords: Methodological terms such as "exploratory factor analysis" and "self-report questionnaire" must be removed and replaced with precise thematic descriptors.

Conclusion: In its current form, the manuscript does not meet the standards for publication. A substantial revision is required to remove irrelevant theoretical information and to clarify exactly what the instrument measures, while formally addressing the limitations of self-report data regarding social desirability bias.

**********

what does this mean?). If published, this will include your full peer review and any attached files.

If you choose “no”, your identity will remain anonymous but your review may still be made public.

**Do you want your identity to be public for this peer review?**  For information about this choice, including consent withdrawal, please see our Privacy Policy

Reviewer #3: No

**********

[NOTE: If reviewer comments were submitted as an attachment file, they will be attached to this email and accessible via the submission site. Please log into your account, locate the manuscript record, and check for the action link "View Attachments". If this link does not appear, there are no attachment files.]

To ensure your figures meet our technical requirements, please review our figure guidelines: https://journals.plos.org/plosone/s/figures

You may also use PLOS’s free figure tool, NAAS, to help you prepare publication quality figures: https://journals.plos.org/plosone/s/figures#loc-tools-for-figure-preparation.

NAAS will assess whether your figures meet our technical requirements by comparing each figure against our figure specifications.

# Author comment: Author response to Decision Letter 4

**DOI:** 10.1371/journal.pone.0355044.r008

18 May 2026

All the reviewer’s comments have been addressed in the revised version of the manuscript.

Attachment Submitted filename: Response_to_Reviewers_auresp_4.docx

# Peer review: Decision Letter 4

**DOI:** 10.1371/journal.pone.0355044.r009

**Authors:** Leire Aperribai

26 Jun 2026

Design, Development, and Pilot Study of a Scale for Educational Professionals to Assess Theory of Mind Skills Regarding Children with ASD.

PLOS One

Dear Dr. Soubelet,

In this 4th revision of the manuscript, reviewer 3 has considered a minor revision. Please, take into account and fulfill the requirements consciensciously to continue with the revision procedure.

Please include the following items when submitting your revised manuscript:

- A letter that responds to each point raised by the academic editor and reviewer(s). You should upload this letter as a separate file labeled 'Response to Reviewers'.
- A marked-up copy of your manuscript that highlights changes made to the original version. You should upload this as a separate file labeled 'Revised Manuscript with Track Changes'.
- An unmarked version of your revised paper without tracked changes. You should upload this as a separate file labeled 'Manuscript'.

If you would like to make changes to your financial disclosure, please include your updated statement in your cover letter. Guidelines for resubmitting your figure files are available below the reviewer comments at the end of this letter.

If applicable, we recommend that you deposit your laboratory protocols in protocols.io to enhance the reproducibility of your results. Protocols.io assigns your protocol its own identifier (DOI) so that it can be cited independently in the future. For instructions see: https://journals.plos.org/plosone/s/submission-guidelines#loc-laboratory-protocols. Additionally, PLOS ONE offers an option for publishing peer-reviewed Lab Protocol articles, which describe protocols hosted on protocols.io. Read more information on sharing protocols at https://plos.org/protocols?utm_medium=editorial-email&utm_source=authorletters&utm_campaign=protocols.

As the corresponding author, your ORCID iD is verified in the submission system and will appear in the published article. PLOS supports the use of ORCID, and we encourage all coauthors to register for an ORCID iD and use it as well. Please encourage your coauthors to verify their ORCID iD within the submission system before final acceptance, as unverified ORCID iDs will not appear in the published article. *Only*  the individual author can complete the verification step; PLOS staff *cannot*  verify ORCID iDs on behalf of authors.

We look forward to receiving your revised manuscript.

Kind regards,

Leire Aperribai

Academic Editor

PLOS One

**Journal Requirements:**

1. If the reviewer comments include a recommendation to cite specific previously published works, please review and evaluate these publications to determine whether they are relevant and should be cited. There is no requirement to cite these works unless the editor has indicated otherwise.

2. Please review your reference list to ensure that it is complete and correct. If you have cited papers that have been retracted, please include the rationale for doing so in the manuscript text, or remove these references and replace them with relevant current references. Any changes to the reference list should be mentioned in the rebuttal letter that accompanies your revised manuscript. If you need to cite a retracted article, indicate the article’s retracted status in the References list and also include a citation and full reference for the retraction notice.

[Note: HTML markup is below. Please do not edit.]

Reviewers' comments:

Reviewer's Responses to Questions

**Comments to the Author**

Reviewer #3: (No Response)

**********

2. Is the manuscript technically sound, and do the data support the conclusions?

Reviewer #3: Partly

**********

3. Has the statistical analysis been performed appropriately and rigorously?

Reviewer #3: N/A

**********

4. Have the authors made all data underlying the findings in their manuscript fully available?

The PLOS Data policy

Reviewer #3: No

**********

5. Is the manuscript presented in an intelligible fashion and written in standard English?

Reviewer #3: Yes

**********

Reviewer #3: I appreciate the substantial improvements made throughout the review process. The manuscript is now clearer and more consistent in terms of its objectives, theoretical framework, interpretation of findings, and discussion of limitations.

However, some conceptual ambiguity remains regarding the distinction between Theory of Mind itself and self-reported ToM-related skills. I encourage the authors to continue emphasizing this distinction throughout the manuscript and to maintain a cautious interpretation of the findings given the exploratory nature of the study.

**********

what does this mean?). If published, this will include your full peer review and any attached files.

If you choose “no”, your identity will remain anonymous but your review may still be made public.

**Do you want your identity to be public for this peer review?**  For information about this choice, including consent withdrawal, please see our Privacy Policy

Reviewer #3: No

**********

[NOTE: If reviewer comments were submitted as an attachment file, they will be attached to this email and accessible via the submission site. Please log into your account, locate the manuscript record, and check for the action link "View Attachments". If this link does not appear, there are no attachment files.]

To ensure your figures meet our technical requirements, please review our figure guidelines: https://journals.plos.org/plosone/s/figures

You may also use PLOS’s free figure tool, NAAS, to help you prepare publication quality figures: https://journals.plos.org/plosone/s/figures#loc-tools-for-figure-preparation.

NAAS will assess whether your figures meet our technical requirements by comparing each figure against our figure specifications.

# Author comment: Author response to Decision Letter 5

**DOI:** 10.1371/journal.pone.0355044.r010

6 Jul 2026

No comments

Attachment Submitted filename: Response_to_Reviewers_auresp_5.docx

# Editor report: Decision Letter 5

**DOI:** 10.1371/journal.pone.0355044.r011

**Authors:** Leire Aperribai

17 Jul 2026

Design, Development, and Pilot Study of a Scale for Educational Professionals to Assess Theory of Mind-Related Skills Regarding Children with ASD.

PONE-D-25-22255R5

Dear Carolina Messer-Soubelet,

We’re pleased to inform you that your manuscript has been judged scientifically suitable for publication and will be formally accepted for publication once it meets all outstanding technical requirements.

Within one week, you’ll receive an e-mail detailing the required amendments. When these have been addressed, you’ll receive a formal acceptance letter and your manuscript will be scheduled for publication.

An invoice will be generated when your article is formally accepted. Please note, if your institution has a publishing partnership with PLOS and your article meets the relevant criteria, all or part of your publication costs will be covered. Please make sure your user information is up-to-date by logging into Editorial Manager at Editorial Manager® and clicking the ‘Update My Information' link at the top of the page. For questions related to billing, please contact billing support.

If your institution or institutions have a press office, please notify them about your upcoming paper to help maximize its impact. If they’ll be preparing press materials, please inform our press team as soon as possible -- no later than 48 hours after receiving the formal acceptance. Your manuscript will remain under strict press embargo until 2 pm Eastern Time on the date of publication. For more information, please contact onepress@plos.org.

Kind regards,

Leire Aperribai

Academic Editor

PLOS One

# Editor report: Acceptance letter

**DOI:** 10.1371/journal.pone.0355044.r012

**Authors:** Leire Aperribai

PONE-D-25-22255R5

PLOS One

Dear Dr. Messer-Soubelet,

I'm pleased to inform you that your manuscript has been deemed suitable for publication in PLOS One. Congratulations! Your manuscript is now being handed over to our production team.

At this stage, our production department will prepare your paper for publication. This includes ensuring the following:

* All references, tables, and figures are properly cited

* All relevant supporting information is included in the manuscript submission,

* There are no issues that prevent the paper from being properly typeset

You will receive further instructions from the production team, including instructions on how to review your proof when it is ready. Please keep in mind that we are working through a large volume of accepted articles, so please give us a few days to review your paper and let you know the next and final steps.

Lastly, if your institution or institutions have a press office, please let them know about your upcoming paper now to help maximize its impact. If they'll be preparing press materials, please inform our press team within the next 48 hours. Your manuscript will remain under strict press embargo until 2 pm Eastern Time on the date of publication. For more information, please contact onepress@plos.org.

You will receive an invoice from PLOS for your publication fee after your manuscript has reached the completed accept phase. If you receive an email requesting payment before acceptance or for any other service, this may be a phishing scheme. Learn how to identify phishing emails and protect your accounts at https://explore.plos.org/phishing.

If we can help with anything else, please email us at customercare@plos.org.

Thank you for submitting your work to PLOS One and supporting open access.

Kind regards,

PLOS ONE Editorial Office Staff

on behalf of

Dr. Leire Aperribai

Academic Editor

PLOS One
